# Role of primary and secondary care data in atrial fibrillation ascertainment: impact on risk factor associations, patient management, and mortality in UK Biobank

**DOI:** 10.1093/europace/euae291

**Published:** 2025-02-06

**Authors:** C Fielder Camm, Adam Von Ende, Parag R Gajendragadkar, Guilherme Pessoa-Amorim, Marion Mafham, Naomi Allen, Sarah Parish, Barbara Casadei, Jemma C Hopewell

**Affiliations:** Nuffield Department of Population Health, University of Oxford, Big Data Institute, Old Road Campus, Oxford OX3 7LF, UK; Nuffield Department of Population Health, University of Oxford, Big Data Institute, Old Road Campus, Oxford OX3 7LF, UK; Nuffield Department of Population Health, University of Oxford, Big Data Institute, Old Road Campus, Oxford OX3 7LF, UK; Nuffield Department of Population Health, University of Oxford, Big Data Institute, Old Road Campus, Oxford OX3 7LF, UK; Nuffield Department of Population Health, University of Oxford, Big Data Institute, Old Road Campus, Oxford OX3 7LF, UK; Nuffield Department of Population Health, University of Oxford, Big Data Institute, Old Road Campus, Oxford OX3 7LF, UK; Nuffield Department of Population Health, University of Oxford, Big Data Institute, Old Road Campus, Oxford OX3 7LF, UK; Division of Cardiovascular Medicine, Radcliffe Department of Medicine, University of Oxford, Oxford, UK; Nuffield Department of Population Health, University of Oxford, Big Data Institute, Old Road Campus, Oxford OX3 7LF, UK

**Keywords:** Atrial fibrillation, Electronic healthcare records, Primary care, Ascertainment, UK Biobank

## Abstract

**Aims:**

Electronic healthcare records (EHR) are at the forefront of advances in epidemiological research emerging from large-scale population biobanks and clinical studies. Hospital admissions, diagnoses, and procedures (HADP) data are often used to identify disease cases. However, this may result in incomplete ascertainment of chronic conditions such as atrial fibrillation (AF), which are principally managed in primary care (PC). We examined the relevance of EHR sources for AF ascertainment, and the implications for risk factor associations, patient management, and outcomes in UK Biobank.

**Methods and results:**

UK Biobank is a prospective study, with HADP and PC records available for 230 000 participants (to 2016). AF cases were ascertained in three groups: from PC records only (PC-only), HADP only (HADP-only), or both (PC + HADP). Conventional statistical methods were used to describe differences between groups in terms of characteristics, risk factor associations, ascertainment timing, rates of anticoagulation, and post-AF stroke and death. A total of 7136 incident AF cases were identified during 7 years median follow-up (PC-only: 22%, PC + HADP: 49%, HADP-only: 29%). There was a median lag of 1.3 years between cases ascertained in PC and subsequently in HADP. AF cases in each of the ascertainment groups had comparable baseline demographic characteristics. However, AF cases identified in hospital data alone had a higher prevalence of cardiometabolic comorbidities and lower rates of subsequent anticoagulation (PC-only: 44%, PC + HADP: 48%, HADP-only: 10%, *P* < 0.0001) than other groups. HADP-only cases also had higher rates of death [PC-only: 9.3 (6.8, 12.7), PC + HADP: 23.4 (20.5, 26.6), HADP-only: 81.2 (73.8, 89.2) events per 1000 person-years, *P* < 0.0001] compared to other groups.

**Conclusion:**

Integration of data from primary care with that from hospital records has a substantial impact on AF ascertainment, identifying a third more cases than hospital records alone. However, about a third of AF cases recorded in hospital were not present in the primary care records, and these cases had lower rates of anticoagulation, as well as higher mortality from both cardiovascular and non-cardiovascular causes. Initiatives aimed at enhancing information exchange of clinically confirmed AF between healthcare settings have the potential to benefit patient management and AF-related outcomes at an individual and population level. This research underscores the importance of access and integration of de-identified comprehensive EHR data for a definitive understanding of patient trajectories, and for robust epidemiological and translational research into AF.

## Graphical Abstract

**Graphical Abstract:**
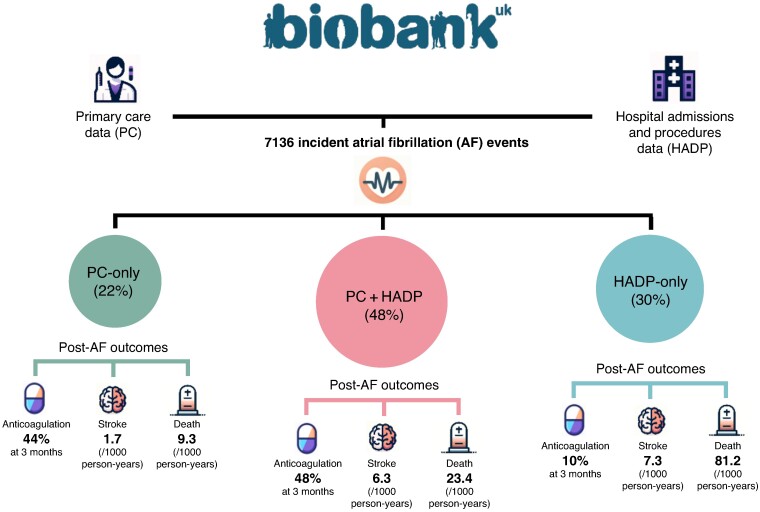

**What’s new?**

- Large-scale electronic healthcare data in UK Biobank participants demonstrates that the healthcare setting in which atrial fibrillation is ascertained has implications for patient management and outcomes.
- Integrating primary care data with hospital records identifies nearly a third more AF cases versus hospital data alone, and also identifies AF earlier.
- Patients with AF identified through hospital records alone have more pre-existing cardiometabolic comorbidities, lower anticoagulation rates, and higher mortality than those recorded in primary care.
- Initiatives aimed at improving data interoperability across healthcare settings should be prioritized in order to optimize patient care.

## Introduction

Electronic healthcare records (EHR) have played a pivotal role in the development and success of large-scale biobanks, and have enabled a range of high-impact epidemiological studies with wide-ranging applications.^1–3^ In general, biobanks have utilized record linkage to hospital admissions and mortality data to ascertain disease status.^4–7^ However, for chronic conditions such as atrial fibrillation (AF), which are often diagnosed and managed in the community, omission of primary care data will lead to incomplete ascertainment and potential biases in disease representation.

AF is the most common sustained cardiac arrhythmia,^8^ and is associated with significant mortality and morbidity burden.^9^ Hospital admissions in those with AF may be due to AF-related symptoms,^10^ comorbidities or outcomes,^11^ or be entirely unrelated to AF. In the UK, AF is not recorded as the primary reason for hospitalization in ∼50% of cases,^12^ and in up to 80% of cases in other European countries.^13^ Consequently, relying solely on secondary care data for identifying AF can lead to incomplete case ascertainment and patient misclassification. This has the potential to result in a range of biases (e.g. information, attribution, and selection biases) that affect the reliability of studies of AF examining risk factors, management and sequelae, as well as limit their generalizability.

In the UK, the majority of medical care is provided through the National Health Service, which delivers a comprehensive range of services that are typically accessed following assessment in primary care. Routine referrals from primary to secondary care are made on the basis of clinical criteria and the need for more complex investigations and specialist treatment plans. Clinical diagnoses made in primary care often result from routine health checks, monitoring of patients with risk factors, and first-line investigations of symptoms (e.g. physical examination, blood tests, and 12-lead ECG). As a consequence, many chronic conditions are diagnosed in the community, with an estimated 45% of AF cases first recorded in primary care.^14,15^ AF diagnoses can also be recorded in secondary care when patients present to hospital for a variety of reasons, such as admission for an unrelated complaint, surgical assessment or procedure, or a related or unrelated emergency admission. Any diagnosis recorded in secondary care (whether or not the main reason for admission) should be routinely communicated to the primary care provider to ensure appropriate monitoring and long-term management, as well as to maintain an accurate patient record. Given the different emphasis of these routes to diagnosis, individuals with AF first recorded in hospital may be more acutely ill and have more comorbidities, as well as have their AF diagnosis considerably delayed when compared to those initially recorded in primary care.^10,16^ Furthermore, this highlights the importance of establishing a robust understanding of the relevance of the healthcare setting in which AF diagnoses are recorded, and any impact it may have on patient management and outcomes.

UK Biobank (UKB) is a world-leading deeply phenotyped resource for large-scale epidemiology and population health research.^17^ This study aims to leverage EHR data available in the UKB population to evaluate whether identification of AF cases varies between primary care and hospital admissions diagnoses and procedures data, and explore potential systematic differences in the cases ascertained using different data sources in terms of patient characteristics, timing of ascertainment, patient management (i.e. anticoagulation), and outcomes including stroke and death.

## Methods

### Study population

UK Biobank is a prospective study of over 500 000 British individuals, aged 40–69 years when recruited between 2006 and 2010.^18^ UK Biobank has extensive phenotypic and health-related information from standardized questionnaires, physical and biological measurements, genotyping and imaging studies, and ongoing linkage to EHR, including primary and secondary care data. UK Biobank primary care data include coded clinical events (e.g. diagnoses, laboratory results, and procedures), prescription records, and administrative codes. UK Biobank secondary care data include hospital inpatient data, and associated diagnostic and procedure codes, and information related to admission and discharge. Secondary healthcare records are available for all participants, however only 230 060 participants (as of June 2024) also have linkage to their primary care records.^17^ This study is restricted to individuals with linkage to both primary and secondary care records available.

### Ascertainment of AF cases

AF hospitalizations were ascertained using International Classification of Disease 10 (ICD-10) codes and OPCS Classification of Interventions and Procedures (OPCS-4) codes (see Supplementary material online, *Table S1*) from hospital admissions databases [Hospital Episode Statistics (HES; England), Patient Episode Database for Wales (PEDW; Wales), and Scottish Morbidity Record (SMR; Scotland)]. These codes have been used to identify AF in previous studies.^4,19^

AF primary care cases were derived by systematically mapping ICD-10 and OPCS-4 codes to Read Codes (versions 2 and 3), using NHS TRUD mapping files (see Supplementary material online, *Figure S1*).^20,21^ Additional Read Codes were identified through the use of a keyword search of Read Code descriptions (keywords: ‘atrial fibrillation’ and ‘atrial flutter’). All Read Codes obtained through ICD-10/OPCS-4 mapping and the keyword search were verified by a clinician before confirming the final code list. A list of Read Version 2 and Read Version 3 (CTV3) codes used to identify AF, and their mappings to ICD-10/OPCS-4, is provided in Supplementary material online, *Table S2*.

In total, 110 primary care Read Codes relating to AF diagnoses were identified. Of these, most (*n* = 88; 80%) suggest ascertainment of AF during the current care encounter, while the remainder (*n* = 22; 20%) refer to monitoring of pre-existing AF or suggest confirmation of a prior diagnosis of AF. A majority of the 110 codes (*n* = 69, 63%) was identified by mapping from ICD-10/OPCS-4, with the remainder identified by keyword search (see Supplementary material online, *Table S2*).

Prevalent AF cases were determined on the basis of self-reported AF at the baseline UKB assessment, or AF ascertained using available HADP or primary care records pre-dating recruitment. Incident AF cases were determined based on the presence of a HADP or primary care record of AF after date of UKB recruitment (in those with no history of AF) until the end of data availability (31 May 2016 for participants in England, 31 October 2016 for Scotland, and 29 February 2016 for Wales; Supplementary material online, *Table S3*). Individuals with AF events lacking corresponding event dates were excluded, as their status as prevalent or incident cases could not be determined. Incident AF cases were then divided into three ascertainment groups, those identified: only in primary care records (PC-only); in both primary care and HADP records (PC + HADP); and only in HADP records (HADP-only; Supplementary material online, *Figure S2*).

### Participant characteristics

Participant characteristics, self-reported disease history, prevalent medication use (recorded at a nurse-led interview), and the CHARGE-AF clinical risk score^22^ were defined at recruitment, and an AF polygenic risk score (PRS) estimated.^23^ Further description is provided in the Supplementary Methods.

### Anticoagulation status in AF cases

To examine differences in stroke risk management based on the source of AF ascertainment, anticoagulation status subsequent to first ascertainment of AF was determined. Anticoagulants were identified from the British National Formulary (BNF),^24^ and extracted from primary care data using BNF codes, drug names, or Read 2 codes (see Supplementary material online, *Table S4*). Based on the available prescription data, a participant was deemed to be anticoagulated if they were prescribed anticoagulation at the time of, or any time after, their AF ascertainment. In accordance with guidelines for initiating oral anticoagulation in AF patients since 2012,^14,25^ analyses were limited to those with a baseline CHA_2_DS_2_-VA score (≥2) that would warrant anticoagulation.^26^

### Ethics approval

This research has been conducted using the UK Biobank Resource under application number 14568. All procedures and data collection in UK Biobank were approved by the UK Biobank Research Ethics Committee (reference number 11/NW/0274) with participants providing full written informed consent for participation in UK Biobank and subsequent use of their data for approved applications.

### Statistical analyses

Continuous variables were described using mean (standard deviation; SD) and median (interquartile range; IQR), where appropriate, and categorical variables were described using counts (%). Differences between ascertainment groups were assessed using one-way analysis of variance (ANOVA) for continuous variables and χ^2^ tests for categorical variables. Cumulative incidence curves illustrating time to a HADP AF record subsequent to a primary care AF record, and vice versa, were examined to assess the time course of AF ascertainment. Multinomial logistic regression models were used to estimate odds ratios describing the association of clinical and genetic risk factors with incident AF by ascertainment group (No AF, PC-only, PC + HADP, HADP-only), and were adjusted for service usage (the total number of hospital admissions and the total number of primary care encounters). Poisson regression models were used to estimate incidence of post-AF stroke and death per 1000 person-years at risk, where time-at-risk began at ascertainment of AF (second ascertainment for PC + HADP individuals). Models were adjusted in stages as follows: (i) no covariates, (ii) baseline risk factors (age, sex, ethnicity, Townsend Deprivation Index, body mass index, smoking, alcohol use, hypertension, type 2 diabetes, heart failure, and myocardial infarction) and service usage, and (iii) baseline risk factors, service usage, and the Charlson Comorbidity Index at the time of AF ascertainment. Further details on variable definitions and methodology are provided in the Supplementary Methods. All analyses were performed using SAS 9.4^27^ and R version 4.1.^28^

## Results

### Ascertainment of prevalent AF cases

Among 230 050 UKB participants, 4374 (1.9%) prevalent AF cases were identified (see Supplementary material online, *Figure S3*). There was moderate overlap between self-reported, HADP, and primary care ascertained AF, with 60% of prevalent cases identified in at least two sources. The use of primary care data identified 706 (16.1%) prevalent AF cases not identified using HADP or self-reported information.

### Ascertainment of incident AF cases

In the 225 676 participants without prevalent AF, 7136 incident AF cases (3.2% of participants) were identified during a median of 7 years of follow-up (IQR 6.6–8.0). The mean age of AF cases was 62 years (SD = 6; range = 38–71), and 2671 (37%) were female. Of the AF cases, 5032 (70.5%) had at least one primary care record of AF, 5565 (78.0%) had at least one HADP record of AF, and 3461 (48.5%) had both a primary care and HADP AF record. Hence, 1571 (22.0%) of cases were categorized as PC-only, 2104 (29.5%) as HADP-only, and 3461 (48.5%) as PC + HADP. Of those in the PC + HADP group, 1598 (46.2%) were first recorded in primary care and 1205 (34.8%) in HADP, while 658 (19.0%) had their AF recorded on the same day in both sources.

Of 5032 cases with a primary care record of AF, the majority (57.7%) was ascertained via Read Code ‘G5730’ for ‘Atrial Fibrillation’ (see Supplementary material online, *Table S5*). Of 5565 cases with a HADP record of AF, almost all (97.8%) were identified using ICD-10 codes, with only 2.2% identified using OPCS codes alone.

AF may be detected or recorded when individuals attend medical settings for other reasons (e.g. 84.4% of HADP-only AF cases are recorded in the non-primary diagnostic position), and we therefore examined hospital/primary care resource usage. Of participants in the PC-only group, 1371 (87.3%) had at least one hospital admission for another cause during the study period (median 3, IQR 1–6) with 524 (33.3%) hospitalized for any reason subsequent to AF ascertainment (median = 0, IQR 0–1). Of those in the HADP-only group, 2093 (99.5%) had at least one GP encounter during the study period, with 1772 (84.2%) subsequent to the HADP-based AF ascertainment.

All subsequent analyses are limited to incident AF cases.

### Participant characteristics, by ascertainment source

**Figure 1:**
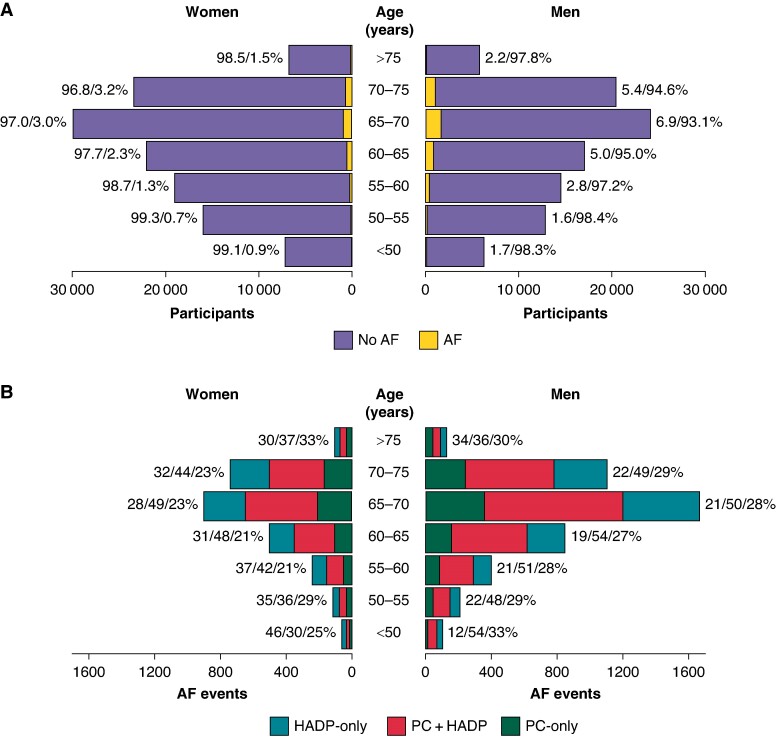
Proportion of participants in each AF ascertainment group, by age at ascertainment and sex. (*A*) The number and proportion of participants with and without AF at the end of follow-up (prevalence), stratified by age and sex. (*B*) The number and proportion of incident AF cases within each ascertainment group (PC-only, PC + HADP, HADP-only), stratified by age at ascertainment and sex. AF, atrial fibrillation; PC, primary care; HADP, hospital admissions and procedures.

Individuals in the three ascertainment groups were similar with respect to age at baseline, ethnicity, body mass index, and alcohol use (*Table 1*). Mean age at baseline was 62 years (SD = 6) and was 66 years (SD = 6) at AF ascertainment in all groups. There was a higher proportion of men in the PC + HADP (65.0%) vs. the PC-only (60.3%) and HADP-only (60.2%) groups (*Table 1* and *Figure 1*). Individuals in the HADP-only group had a higher burden of current smoking, hypertension, type 2 diabetes, medication use, and other cardiovascular comorbidities than individuals in the PC + HADP group, and the latter generally had a higher burden of these risk factors than individuals in the PC-only group (*Table 1*). Consequently, the proportion of participants with a CHA_2_DS_2_-VA score ≥ 2 at baseline (which is prior to AF ascertainment) was slightly higher in the HADP-only (43.6%, *n* = 918) and PC + HADP groups (41.5%, *n* = 1437) than in the PC-only group (36.5%, *n* = 574).

**Table 1:** Baseline participant characteristics by AF ascertainment group (*n* = 225 676; 7136 incident AF cases)

	AF ascertainment group			
Characteristic	PC-only (*n* = 1571)	PC + HADP (*n* = 3461)	HADP-only (*n* = 2104)	*P*-value
Age (years)	61.77 ± 6.13	62.20 ± 5.82	61.63 ± 6.25	0.001
Sex (male)	947 (60.3%)	2251 (65.0%)	1272 (60.3%)	<0.001
Race (white)	1533 (97.6%)	3374 (97.5%)	2030 (96.5%)	0.053
Townsend Deprivation Index	−1.47 ± 2.99	−1.40 ± 3.02	−0.98 ± 3.25	<0.001
BMI (kg/m^2^)	28.55 ± 5.16	29.29 ± 5.46	28.86 ± 5.33	<0.001
Current smoker	130 (8.3%)	345 (10.0%)	311 (14.9%)	<0.001
Current drinker	1457 (92.7%)	3168 (91.5%)	1881 (89.4%)	0.001
CHA_2_DS_2_-VA score ≥ 2	574 (36.5%)	1437 (41.5%)	918 (43.6%)	<0.001
HAS-BLED score ≥ 3	327 (20.8%)	862 (24.9%)	509 (24.2%)	0.006
Hand grip strength (kg)	32.32 ± 11.02	32.25 ± 11.21	30.37 ± 11.30	<0.001
Self-reported prior disease				
Hypertension	659 (41.9%)	1638 (47.3%)	956 (45.4%)	0.002
Type 2 diabetes	106 (6.8%)	276 (8.1%)	191 (9.4%)	0.019
Heart failure	8 (0.5%)	17 (0.5%)	11 (0.5%)	0.99
Myocardial infarction	75 (4.8%)	260 (7.5%)	188 (8.9%)	<0.001
Stroke	36 (2.3%)	109 (3.1%)	101 (4.8%)	<0.001
Self-reported medication use				
Cholesterol-lowering	471 (30.0%)	1134 (32.8%)	767 (36.6%)	<0.001
Antihypertensive	547 (34.9%)	1346 (39.0%)	818 (39.0%)	0.013
Anticoagulant	24 (1.5%)	67 (1.9%)	82 (3.9%)	<0.001
Aspirin	407 (25.9%)	1000 (28.9%)	611 (29.1%)	0.058

Values are presented as mean ± standard deviation, number (per cent), or median (interquartile range). *P*-values were calculated using one-way ANOVA for continuous variables and Pearson’s χ^2^ test for categorical variables. Higher Townsend scores indicate greater levels of deprivation. Hand grip strength is the mean of measurements from both the right and left hands.

AF, atrial fibrillation; PC, primary care; HADP, hospital admissions diagnoses and procedures; BMI, body mass index; kg/m^2^, kilograms per metre squared.

### Risk factor associations, by ascertainment source

**Figure 2:**
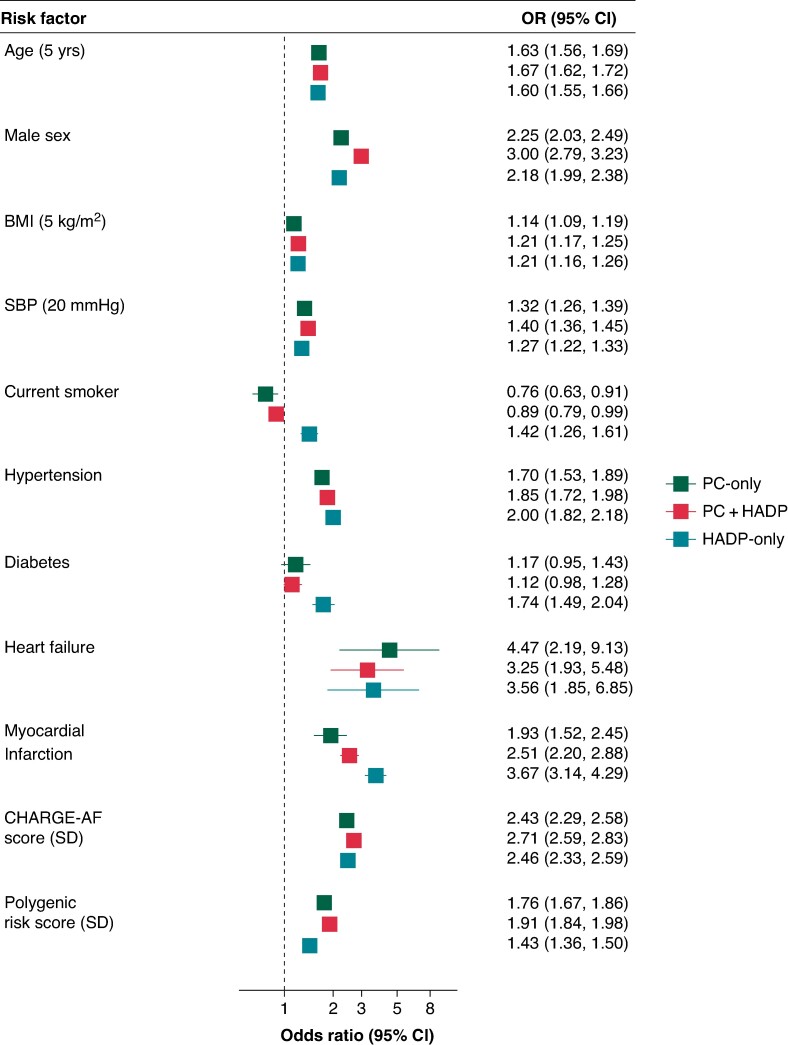
Association of AF risk factors with incident AF, by ascertainment group. Results are based on multinomial logistic models with ‘no AF’ specified as the reference category, adjusted for the total number of primary care interactions and hospitalizations. PC, primary care; HADP, hospital admissions diagnoses and procedures; SD, standard deviation; yrs, years; kg/m^2^, kilograms per metre squared; mmHg, millimetres of mercury; BMI, body mass index; SBP, systolic blood pressure.

Baseline AF risk factors including age, body mass index, hypertension, and heart failure showed comparable risk factor associations with AF across the three ascertainment groups (*Figure 2*). In contrast, history of type 2 diabetes and myocardial infarction were associated with higher risks of AF in the HADP-only group compared to the other groups (all *P* < 0.001). Composite clinical AF risk (based on CHARGE-AF) and genetic risk (based on an AF PRS) were associated with 2.4–2.7-fold and 1.4–1.8-fold higher risks (per SD) respectively across the three ascertainment groups, with the weakest association of the AF PRS observed in the HADP-only group (HADP-only vs. compared to the other groups combined, *P* < 0.001).

### Time course of AF records, by ascertainment source

**Figure 3:**
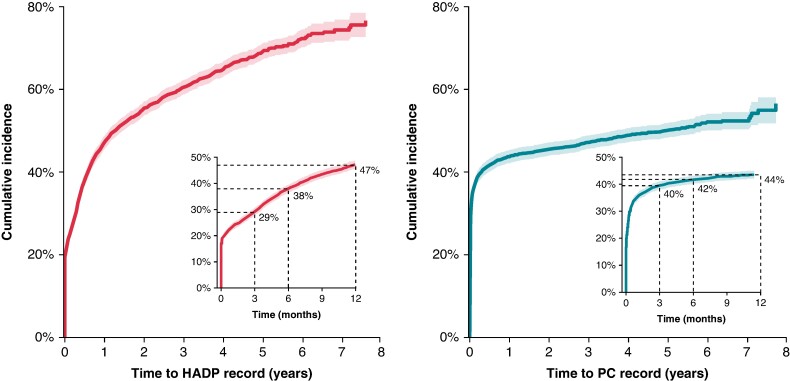
Time from AF ascertainment in one source to ascertainment in the alternate source. Cumulative incidence curves show the time to subsequent AF ascertainment in HADP for those first identified in primary care (left) and the time to subsequent AF ascertainment in primary care for those first identified in HADP (right). Individuals without a subsequent ascertainment in the alternative source are censored at the end of follow-up. Inset plots display incidence and proportions identified at 3, 6, and 12 months. Analyses exclude participants with prior ascertainment in the alternate source.

We investigated the lag time between identification of AF cases in different sources. After excluding 1205 individuals with a HADP record of AF before the primary care record, 3827 individuals with a primary care record of AF went on to have a subsequent HADP record of AF (*n* = 2256, 58.9%) during the follow-up period. Of the former, 17.2% (*n* = 661) had a HADP record on the same day as the primary care record, and a further 7.3% (*n* = 278) had a subsequent HADP record within 30 days of the primary care record. Overall, the median delay between first primary care record of AF and subsequent ascertainment in HADP was 1.3 years (*Figure 3*). The median lag time was longer in women than in men (1.6 vs. 1.1 years; *P* = 0.01), but similar across age groups (*P* = 0.18; Supplementary material online, *Figures S4* and *S5*).

After excluding 1598 individuals with a primary care record of AF before the HADP record, 3967 individuals with a HADP record went on to have a subsequent primary care record of AF (*n* = 1863, 46.9%) during follow-up. Of the former, 16.6% (*n* = 658) had AF identified in a primary care record on the same day as the HADP record, and a further 18.6% (*n* = 737) had a subsequent primary care record within 30 days of the HADP record. Forty-four per cent were recorded in primary care within 1 year. However, the remaining participants had long delays (or were censored), such that the median delay before AF was subsequently ascertained in primary care was 4.9 years. There were no differences in the lag time by sex (*P* = 0.24) or age (*P* = 0.72).

### Resource usage after AF ascertainment

We investigated subsequent resource encounters to consider the opportunities for AF to be recorded after the index event. The majority of HADP-only AF cases had at least one subsequent primary care encounter (84%), and about a third had a subsequent hospital admission (32.7%) (see Supplementary material online, *Table S6*). HADP-only cases were less likely to have AF coded in the primary diagnostic position in the index report compared to other PC + HADP cases (HADP-only: 18.6% vs. PC + HADP: 57.2%), suggesting that AF was not the primary reason for hospital admission.

### Patient management: anticoagulation prescriptions, by ascertainment group

Of AF cases with any primary care data available, 96.2% (*n* = 217 241) had at least one recorded prescription in their primary care record. Of participants with incident AF who met current criteria for anticoagulation at baseline (i.e. CHA_2_DS_2_-VA ≥ 2) 60% (1573/2631) were anticoagulated either at AF ascertainment or thereafter (see Supplementary material online, *Table S7*, Supplementary material online, *Figures S6* and *S7*).

**Figure 4:**
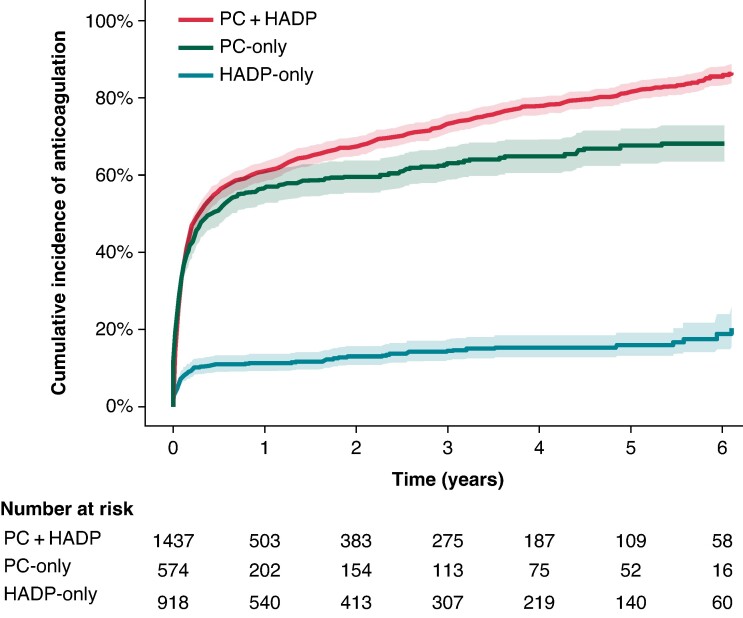
Time to post-AF anticoagulation prescription by ascertainment group. Cumulative incidence curves show the time to first oral anticoagulation prescription after AF ascertainment, stratified by ascertainment group. The analysis is limited to participants with a baseline CHA_2_DS_2_-VA score ≥ 2 where oral anticoagulation is recommended. HADP, hospital admissions diagnoses and procedures; PC, primary care.

Anticoagulation rates differed markedly between ascertainment groups (*Figure 4*). Those in the HADP-only group were less likely to be anticoagulated 3 months post-ascertainment in the community setting (10%) compared with PC + HADP (48%) or PC-only (44%) cases (see Supplementary material online, *Table S7*). This pattern remained consistent across sex, period of ascertainment, analysis restricted to individuals with a Charlson Index of 0 at AF ascertainment, and when considering only AF-related hospital admissions where AF was reported as the primary cause of admission (see Supplementary material online, *Figures S8*–S11).

### Patient outcomes: stroke and death rates, by ascertainment source

**Figure 5:**
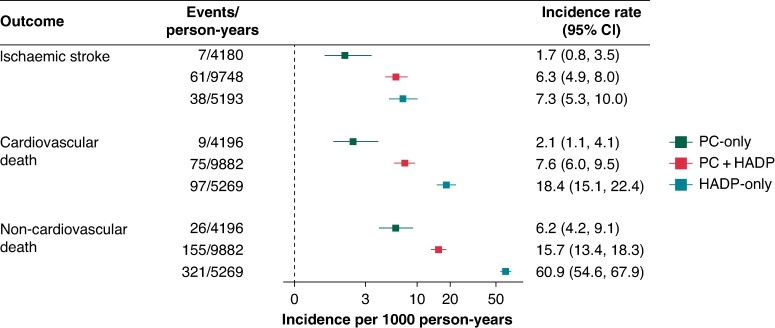
Rates of post-AF stroke and death by ascertainment group. Results are based on Poisson regression models with log of time-at-risk as an offset. Time-at-risk begins at AF ascertainment (second AF ascertainment for PC + HADP individuals). PC, primary care; HADP, hospital admissions diagnoses and procedures; CI, confidence interval.

We estimated rates of ischaemic stroke and cardiovascular and non-cardiovascular death after AF by ascertainment group. AF cases in the PC + HADP and HADP-only groups had similar rates of subsequent ischaemic stroke per 1000 person-years at risk (6.3, 95% CI: 4.9, 8.0 vs. 7.3, 95% CI: 5.3, 10.0), in contrast to the lower rates in the PC-only group (1.7, 95% CI: 0.8, 3.5; *Figure 5*). This pattern was consistent after adjusting for baseline risk factors and service usage, and also when further adjusted for the Charlson Comorbidity Index at time of initial AF ascertainment (see Supplementary material online, *Table S8*).

AF cases in the HADP-only group had higher rates of cardiovascular death per 1000 person-years (18.4, 95% CI: 15.1, 22.4) than cases in the PC + HADP (7.6, 95% CI: 6.0, 9.5) and PC-only groups (2.1, 95% CI: 1.1, 4.1). AF cases in the HADP-only group had substantially higher rates of non-cardiovascular death per 1000 person-years (60.79, 95% CI: 54.46, 67.79) than cases in the PC + HADP (15.7, 95% CI: 13.4, 18.3) and PC-only groups (6.2, 95% CI: 4.2, 9.1). This pattern was consistent after adjusting for baseline risk factors and Charlson Comorbidity Index at time of initial AF ascertainment, although the incidence rate in the HADP-only group after adjustment was attenuated (31.78, 95% CI: 27.2, 37.2; Supplementary material online, *Table S8*).

## Discussion

Linkage of hospital admissions to bespoke participant data has revolutionized the organization and follow-up of large-scale cohorts for epidemiological research.^29^ However, reliance on hospital admissions alone for the identification of chronic conditions can limit ascertainment.^14,15^ Our study aimed to compare and contrast AF cases ascertained through primary care and HADP data in the UKB and consider its wider relevance. The use of primary care data identified over 28% more incident AF cases than HADP data alone. HADP-only AF cases had slightly higher levels of baseline cardiovascular disease and medication use compared to those in the PC-only or PC + HADP groups. In addition, some AF risk factors showed stronger associations with HADP-only AF cases. AF cases only identified via HADP data had far lower rates of anticoagulation (albeit largely similar rates of subsequent stroke), and had much higher rates of death, particularly non-cardiovascular death, compared to those identified in primary care data alone.

### Additional AF cases identified through primary care

The considerable additional yield of incident AF cases through inclusion of primary care data (28%) suggests that integration of primary care and hospital admissions for AF ascertainment will have material advantages for detecting epidemiological associations. This is somewhat larger than the 18% reported in a previous study integrating primary and secondary care data available up to 2010,^15^ which may be partly attributed to the introduction of AF into the Quality and Outcomes Framework in 2006, which incentivized detection of AF in primary care.^30^ Overall, this highlights the importance of primary care data to ensure more complete ascertainment of AF.

### Participant characteristics and risk factor associations

AF is associated with age, male sex, and a wide range of comorbidities including obesity, heart failure, and stroke.^8^ These conditions are also generally associated with higher rates of hospital admissions,^31–33^ and therefore AF cases ascertained from hospital records are likely to represent a more diseased and frail population. Consistent with previous reports,^15^ risk factor associations with age, sex, body mass index, and blood pressure were broadly similar across groups in the present study, albeit slightly stronger associations of type 2 diabetes and myocardial infarction with AF were observed in the HADP-only group, suggesting a higher degree of cardiometabolic comorbidity up to 10 years (given length of follow-up available) prior to AF ascertainment. Risk factor associations were adjusted for the number of hospitalizations to mitigate potential differences arising from ascertainment bias that may arise given patients with comorbidities are more frequently hospitalized. However, the impact of such biases on epidemiological associations cannot be eliminated, and further emphasizes the need for a comprehensive understanding of AF arising in different healthcare settings.

The higher rates of non-cardiovascular death in the HADP-only group may indicate a higher degree of underlying frailty/comorbidity in this group that is not explained by baseline cardiovascular risk factors. Interestingly, the AF PRS was associated with lower risk of AF in the HADP-only group compared to the other groups; which could have implications for genetic studies (e.g. GWAS) of AF, which commonly rely on HADP data sources alone when capitalizing on data from population biobanks.

### Timing of AF ascertainment

AF is commonly diagnosed in the community,^14,15,34^ and this can lead to a delay between primary care and HADP-based ascertainment. Among AF cases identified using primary care data, there was a median delay of 1.3 years before AF was recorded in the HADP data, consistent with data available up to 2010.^15^ This delay in ascertainment using HADP sources may result in ascertainment bias at baseline, and supports excluding the initial few years of follow-up in prospective studies that examine exposure–outcome associations using HADP-defined AF, to mitigate potential reverse causation.

### Information exchange between healthcare settings

NHS guidance indicates that patient diagnoses recorded in the hospital setting should be transcribed to the primary care setting in a timely and responsive manner so that primary care providers can make informed decisions about patient care.^35,36^ Therefore, the substantial number of AF cases only recorded in HADP may indicate inadequate ‘handover’ of diagnostic information. Recent studies on information transfer between the healthcare settings in the UK are limited, however evidence suggests that deficits in information exchange of diagnostic information between hospital-based and primary care settings are common, and may adversely impact clinical outcomes.^37^ Differences in the central coding of diagnoses (that are made available via electronic records and used in this study) and in discharge summaries provided to primary care providers, where inclusion/exclusion of AF may reflect active clinical judgment, may also be possible. Since primary care relies on hospital discharge summaries, when these summaries fail to report AF, there can be no transfer of HADP diagnoses to PC records (or subsequent consideration of anticoagulation).

Several national initiatives are currently underway to enhance the interoperability of primary and secondary coding systems and facilitate seamless information exchange across care settings.^38^ One such initiative, Shared Care Records, leverages standardized clinical vocabularies like SNOMED CT to provide comprehensive patient information accessible across care settings. SNOMED CT, a merger of Read Codes with SNOMED RT, is now recommended as the single terminology system across all care settings in England. While all primary care system suppliers in England have integrated SNOMED CT since 2018 (post-dating data availability in the current study), its adoption in secondary services is ongoing.^38^

### Implications for patient management

Anticoagulation has been shown to reduce the risk of AF-associated stroke.^25,39^ Our results demonstrate that HADP-only AF cases were much less likely to be anticoagulated than PC-only or PC + HADP AF cases. This pattern remained consistent across sensitivity analyses, including when excluding individuals with comorbidities, as especially frail individuals may be less frequently recommended for anticoagulation;^40^ and when including only AF cases for which AF was the primary reason for admission, as an ancillary AF diagnosis may be more prone to ascertainment bias.^41^ Clinical judgement as well as patient choice and individual circumstances may impact on treatment decisions.^42–44^ Nevertheless, the magnitude of the differences in anticoagulation rates observed in this study, and consistency in the pattern across sensitivity analyses, suggests that improvements in provision of anticoagulation (as per guidelines) can be made. Establishing more robust and efficient communication systems of clinically relevant diagnoses between secondary and primary care may further support improved personalized management of AF.

### Implications for AF-related patient outcomes

AF is associated with a higher risk of stroke and with cardiovascular and all-cause mortality.^45–47^ Despite small numbers of strokes, we found that the HADP-only (*n* strokes = 38) and PC + HADP (*n* strokes = 61) groups had similar fully adjusted rates of subsequent stroke. However, these rates were higher compared to the PC-only group (*n* strokes = 7). With respect to subsequent death, the HADP-only group had substantially higher rates of cardiovascular death (>two-fold vs. PC + HADP; eight-fold vs. PC-only) and non-cardiovascular death (>four-fold vs. PC + HADP; 10-fold vs. PC-only). The rate of non-cardiovascular death in the HADP-only group was strongly attenuated after adjustment for the Charlson Comorbidity Index at time of AF ascertainment, suggesting that differences in mortality are somewhat explained by multimorbidity and frailty among HADP-only AF cases (compared to those derived from other sources).

### Strengths and limitations

This study has a number of strengths, including its large sample size and the integration of both extensive phenotypic information from UKB alongside linkage to EHR data, including prescriptions, enabling a comprehensive assessment of the impact of ascertainment source on AF-related epidemiology. In addition, a systematic approach for mapping commonly utilized ICD-10/OPCS-4 codes to primary care diagnostic codes is provided, enabling reproducibility and applicability to more contemporary clinical coding systems such as SNOMED CT.

Despite the breadth of data available, current coding practices in the UK can lead to limitations when utilizing EHR for research purposes. Information on the reliability of PC and HADP data for capturing clinically relevant AF is sparse, although limited validation studies suggest that the positive predictive value in both PC and hospital records may be as high as 98%.^48,49^ In addition, subtypes of AF (e.g. paroxysmal and persistent) are rarely coded but could facilitate a more comprehensive understanding of this arrhythmia. In the present study, primary care and thus prescription data were only available until 2016, limiting our insights into differences between ascertainment sources based on more recent management guidelines (i.e. since the introduction of direct oral anticoagulants, which were not in common usage at that time). Information on dispensing, collection, and patient adherence to medications as well as to hospital-dispended prescriptions were also not available. The absence of access to narrative-based hospital discharge summaries, including unstructured and free-text data, also limits the ability to fully explore information exchange between secondary and primary care, and the role of clinical judgement on the codes reported. Lastly, healthcare systems in other countries may differ in terms of care pathways, coding practices, and EHR coverage, leading to varying implications of AF ascertainment source. However, the integration of data from both primary and secondary care in large-scale biobanks across Europe and the rest of the world^1,50,51^ highlights the potential widespread implications that AF ascertainment source may have on translational epidemiology and our understanding of patient care.

## Conclusions

The integration of primary care data with hospital admissions data identified a third more AF cases. HADP-only cases were more likely to die of cardiovascular and of non-cardiovascular causes, potentially as a result of greater clinical complexity (e.g. advanced frailty, multimorbidity, and progression of disease). These findings suggest that enhancing practices that facilitate timely and complete information exchange of clinically confirmed AF could yield benefits for patient management and AF-related outcomes, both at the individual and population health level. Furthermore, this study highlights the importance of comprehensive access to de-identified electronic healthcare data to facilitate the most robust research into AF and maximize the potential of data for research in UKB and beyond.

## Supplementary Material

euae291_Supplementary_Data

## Data Availability

UK Biobank data are available in accordance with their published data access procedures described at http://www.ukbiobank.ac.uk/using-the-resource/.
