# Supplementary material for: Role of primary and secondary care data in atrial fibrillation ascertainment: impact on risk factor associations, patient management, and mortality in UK Biobank
Source: Europace. 2025 Feb 6;27(2):euae291. doi: 10.1093/europace/euae291 (PMC11799740; doi:10.1093/europace/euae291)
Supplement: euae291_Supplementary_Data [file euae291_supplementary_data.pdf]

Role of primary and secondary care data in atrial fibrillation ascertainment: Impact on risk factor associations, patient management, and mortality in UK Biobank

Supplementary Materials

Contents

Supplementary Materials 1
Supplementary Methods 2
Supplementary Table 1: Codes used to derive the HADP and self-reported AF phenotypes 5
Supplementary Table 2: Codes used to derive the primary care AF phenotype 6
Supplementary Table 3. Country-specific record availability dates 10
Supplementary Table 4: Drug names, Read 2 codes, and BNF codes used to derive anticoagulation status in PC 11
Supplementary Table 5. Frequency of Read v2 and Read v3 codes used to identify 5,032 AF cases through PC records 13
Supplementary Table 6. Additional participant-level information 15
Supplementary Table 7. Proportion of participants who were anticoagulated at 3,6,12 months and at any time after AF ascertainment 17
Supplementary Table 8. Incidence of ischaemic stroke and death after AF ascertainment, by ascertainment group 18
Supplementary Figure 1. Flowchart illustrating the process of mapping the HADP-based AF phenotype to primary care data 19
Supplementary Figure 2. Flowchart of included participants 20
Supplementary Figure 3. Source of prevalent AF ascertainment 21
Supplementary Figure 4. Time from PC to HADP AF ascertainment, by sex and age 22
Supplementary Figure 5: Time from PC to HADP AF ascertainment, by diagnostic code position (AF primary or non-primary) 23
Supplementary Figure 6: Anticoagulation rate at 90 days by year and ascertainment group 24
Supplementary Figure 7: Anticoagulant drug class initiated by year and AF ascertainment group 25
Supplementary Figure 8: Time to anticoagulation grouped by participant sex and stratified by AF ascertainment group 26
Supplementary Figure 9: Time to anticoagulation grouped by date of AF ascertainment and stratified by ascertainment group 27
Supplementary Figure 10: Time to anticoagulation for individuals with a Charlson comorbidity index of 0 at AF ascertainment, stratified by ascertainment group 28
Supplementary Figure 11: Time to anticoagulation for individuals with AF recorded as the primary cause of admission in the hospital record, stratified by ascertainment group 29

Supplementary Methods

Clinical risk scores

The CHARGE-AF score[1] is a composite risk score for 5-year AF prediction. The CHARGE-AF score includes age, race (white), height, weight, systolic and diastolic blood pressure, smoking status (current), anti-hypertensive medication use, type 2 diabetes, heart failure, and myocardial infarction (all determined by baseline self-report in the present study). The CHARGE-AF score is calculated as: 0.508 × age (5 years) + 0.465 × White + 0.248 × height (10 cm) + 0.115 × weight (15 kg) + 0.197 × systolic blood pressure (20 mm Hg) - 0.101 × diastolic blood pressure (10 mm Hg) + 0.359 × current smoker + 0.349 × antihypertensive medication use + 0.237 × type 2 diabetes + 0.701 × congestive heart failure + 0.496 × myocardial infarction.

The CHA_2DS_2-VA score[2] is a clinical prediction rule for estimating the risk of stroke in individuals with AF. The CHA_2DS_2-VA is calculated as a sum of congestive heart failure (1 point), hypertension, age (65-74 years = 1 point, ≥75 = 2 points), type 2 diabetes (1 point), previous stroke or transient ischaemic attack (1 point), and vascular disease (1 point). Comorbidities included in the score were derived from self-report.

AF polygenic risk score

The 'Standard' AF PRS from Genomics PLC was provided as part of the UK Biobank (UKB) Polygenic Risk Score (PRS) Release, which derived PRS for 28 diseases and 25 quantitative traits.[3] The standard AF PRS was derived by applying a modified version of LDPred to meta-analysed summary statistics from three AF GWAS (specifics not reported) comprising a total of 48,646 cases and 256,971 controls, and included a correction for sample overlap from Lin *et al*.[4] Individual PRS values were calculated as the sum of the per-variant posterior effect size multiplied by allele dosage. The 'raw' PRS value was then centred by subtracting out the PRS value predicted from a linear regression of the PRS against the first four components of ancestry, and then standardised by dividing the centred PRS by the ancestry-specific standard deviation. The 'standard' or 'UKB-free' PRS is so-called because it is trained on external data only, in contrast to the 'Enhanced PRS', which is trained on external data and a subset of UKB participants. The Standard AF PRS was validated using data from the 100,000 Genomes Project (100KGP), which showed that the AF PRS had better performance in 100KGP compared to UKB in terms of the magnitude of the association between the PRS and AF risk. The Standard AF PRS also showed favourable performance compared to two other published AF PRS.[3]

HAS-BLED score

The HAS-BLED score[5] is a clinical risk score used to assess the risk of major bleeding in patients with atrial fibrillation who are taking anticoagulant medications. The HAS-BLED score is calculated as a sum of hypertension (1 point), abnormal renal (1 point) or liver (1 point) function, history of stroke (1 point), bleeding history (1 point), labile INR (1 point), age over 65 years (1 point), use of drugs that increase bleeding risk (1 point) and alcohol use (≥8 drinks/week; 1 point). For the present study, the calculated HAS-BLED score does not include unstable/high INRs.

Charlson comorbidity index

The Charlson index is a clinical tool developed to predict 1-year patient mortality using comorbidity data obtained from hospital chart review.[6] The Charlson index score is sum of 19

predefined comorbidities that are assigned weights of 1, 2, 3, or 6. The score (weight) includes myocardial infarction (1), congestive heart failure (1), peripheral vascular disease (1), cerebrovascular disease (1), dementia (1), chronic pulmonary disease (1), connective tissue disease (1), peptic ulcer disease (1), mild liver disease (1), moderate or severe liver disease (3), diabetes without complications (1), diabetes with end-organ damage (2), hemiplegia or paraplegia (2), moderate or severe renal disease (2), any tumour (2), leukaemia (2), lymphoma (2), metastatic solid tumour (6), and AIDS/HIV (6). For the present study, Charlson comorbidities were defined using a validated algorithm for constructing comorbidities based on administrative hospital data.[7]

References

[1] Alonso A, Krijthe BP, Aspelund T, Stepas KA, Pencina MJ, Moser CB, et al. Simple risk model predicts incidence of atrial fibrillation in a racially and geographically diverse population: the CHARGE-AF consortium. *JAMA* 2013; 2: e000102.

[2] Van Gelder IC, Rienstra M, Bunting KV, Casado-Arroyo R, Caso V, Crijns HJ, et al. 2024 ESC Guidelines for the management of atrial fibrillation developed in collaboration with the European Association for Cardio-Thoracic Surgery (EACTS) Developed by the task force for the management of atrial fibrillation of the European Society of Cardiology (ESC), with the special contribution of the European Heart Rhythm Association (EHRA) of the ESC. Endorsed by the European Stroke Organisation (ESO). *European Heart Journal* 2024: ehae176.

[3] Thompson DJ, Wells D, Selzam S, Peneva I, Moore R, Sharp K, et al. UK Biobank release and systematic evaluation of optimised polygenic risk scores for 53 diseases and quantitative traits. *medRxiv* 2022.

[4] Lin DY, Sullivan PF. Meta-analysis of genome-wide association studies with overlapping subjects. *Am J Hum Genet* 2009; 85: 862-872.

[5] Pisters R, Lane DA, Nieuwlaat R, De Vos CB, Crijns HJ, Lip GY. A novel user-friendly score (HAS-BLED) to assess 1-year risk of major bleeding in patients with atrial fibrillation: the Euro Heart Survey. *Chest* 2010; 138: 1093-1100.

[6] Charlson ME, Pompei P, Ales KL, MacKenzie CR. A new method of classifying prognostic comorbidity in longitudinal studies: development and validation. *Journal of chronic diseases* 1987; 40: 373-383.

[7] Quan H, Sundararajan V, Halfon P, Fong A, Burnand B, Luthi J-C, et al. Coding algorithms for defining comorbidities in ICD-9-CM and ICD-10 administrative data. *Medical care* 2005: 1130-1139.

Supplementary Table 1: Codes used to derive the HADP and self-reported AF phenotypes

Code book	Codes
**HADP phenotype**	
ICD10	I48, I480, I481, I482, I483, I484, I489
OPCS-4	K223, K571, K575, K621, K622, K623, K624, K625, X501, X502
**Verbal interview phenotype**	
UKB Code 6	1471, 1483
UKB Code 5	1524, 1553

ICD - International Disease Classification; OPCS-4 - Office of Population Censuses and Surveys Classification of Interventions and Procedures Version 4; UKB - UK Biobank. ICD10 and OPCS codes used to determine disease status from HADP and death registry data. UKB Code 5 and UKB Code 6 used to determine disease status from verbal interview records.

Supplementary Table 2: Codes used to derive the primary care AF phenotype

Read Code	Read Code Type	Read Code Description	Map Type	Mapped ICD-10/OPCS-4 Code	Non-index event
G573.	V2	Atrial fibrillation and flutter	ICD10	I489	No
G5730	V2	Atrial fibrillation	ICD10	I489	No
G5731	V2	Atrial flutter	ICD10	I489	No
G5732	V2	Paroxysmal atrial fibrillation	ICD10	I480	No
G5733	V2	Non-rheumatic atrial fibrillation	ICD10	I489	No
G5734	V2	Permanent atrial fibrillation	ICD10	I489	No
G5735	V2	Persistent atrial fibrillation	ICD10	I481	No
G5736	V2	Paroxysmal atrial flutter	ICD10	I489	No
G5737	V2	Chronic atrial fibrillation	ICD10	I482	No
G5738	V2	Typical atrial flutter	ICD10	I483	No
G5739	V2	Atypical atrial flutter	ICD10	I484	No
G573z	V2	Atrial fibrillation and flutter NOS	ICD10	I489	No
790G2	V2	Percutaneous occlusion of left atrial appendage	OPCS4	K625	No
790G3	V2	Exclusion of left atrial appendage	OPCS4	K223	No
79340	V2	Percutaneous transluminal ablation of atrioventricular node	OPCS4	K571	No
79345	V2	Percutaneous transluminal ablation of atrial wall	OPCS4	K575	No
79348	V2	Percutaneous transluminal ablation of atrial wall NEC	OPCS4	K575	No
793M0	V2	Percutaneous transluminal ablation of pulmonary vein to left atrium conducting system	OPCS4	K621	No
793M1	V2	Percutaneous transluminal ablation of atrial wall for atrial flutter	OPCS4	K622	No
793M2	V2	Percutaneous transluminal internal cardioversion NEC	OPCS4	K624	No
793M3	V2	Percutaneous transluminal ablation of conducting system of heart for atrial flutter NEC	OPCS4	K623	No
7L1H0	V2	Direct current cardioversion	OPCS4	X501	No
7L1H1	V2	External cardioversion NEC	OPCS4	X502	No
7L1H2	V2	Internal electrode cardioversion	OPCS4	K624	No
7L1H3	V2	Electrical sinus rhythm conversion	OPCS4	X501	No
7L1H4	V2	Electrical operative cardiac stimulation	OPCS4	X501	No
7L1H8	V2	Chemical cardioversion	OPCS4	X502	No
14AN.	V2	H/O: atrial fibrillation	KW	NA	Yes
14AR.	V2	History of atrial flutter	KW	NA	Yes

Read Code	Read Code Type	Read Code Description	Map Type	Mapped ICD-10/OPCS-4 Code	Non-index event
212R.	V2	Atrial fibrillation resolved	KW	NA	Yes
3272	V2	ECG: atrial fibrillation	KW	NA	No
3273	V2	ECG: atrial flutter	KW	NA	No
662S.	V2	Atrial fibrillation monitoring	KW	NA	Yes
6A9..	V2	Atrial fibrillation annual review	KW	NA	Yes
7936A	V2	Implantation of intravenous pacemaker for atrial fibrillation	KW	NA	No
8CMW2	V2	Atrial fibrillation care pathway	KW	NA	No
8HTy.	V2	Referral to atrial fibrillation clinic	KW	NA	No
8OAD.	V2	Provision of written information about atrial fibrillation	KW	NA	No
9Os..	V2	Atrial fibrillation monitoring administration	KW	NA	Yes
9Os0.	V2	Atrial fibrillation monitoring first letter	KW	NA	Yes
9Os1.	V2	Atrial fibrillation monitoring second letter	KW	NA	Yes
9Os2.	V2	Atrial fibrillation monitoring third letter	KW	NA	Yes
9Os3.	V2	Atrial fibrillation monitoring verbal invite	KW	NA	Yes
9Os4.	V2	Atrial fibrillation monitoring telephone invite	KW	NA	Yes
9hF..	V2	Exception reporting: atrial fibrillation quality indicators	KW	NA	No
9hF0.	V2	Excepted from atrial fibrillation quality indicators: Patient unsuitable	KW	NA	No
9hF1.	V2	Excepted from atrial fibrillation quality indicators: Informed dissent	KW	NA	No
G573.	V3	Atrial fibrillation and flutter	ICD10	I489	No
G5730	V3	Atrial fibrillation	ICD10	I489	No
G5731	V3	Atrial flutter	ICD10	I489	No
G573z	V3	Atrial fibrillation and flutter NOS	ICD10	I489	No
X202R	V3	Lone atrial fibrillation	ICD10	I489	No
X202S	V3	Non-rheumatic atrial fibrillation	ICD10	I489	No
Xa2E8	V3	Paroxysmal atrial fibrillation	ICD10	I480	No
Xa3rp	V3	Pacer controlled atrial fibril	ICD10	I489	No
Xa7nI	V3	Controlled atrial fibrillation	ICD10	I489	No
XaEga	V3	Rapid atrial fibrillation	ICD10	I489	No
XaOfa	V3	Persistent atrial fibrillation	ICD10	I481	No
XaOft	V3	Permanent atrial fibrillation	ICD10	I489	No
XaaUH	V3	Paroxysmal atrial flutter	ICD10	I489	No
XaeUP	V3	Chronic atrial fibrillation	ICD10	I482	No
XaeUQ	V3	Typical atrial flutter	ICD10	I483	No
XaeUR	V3	Atypical atrial flutter	ICD10	I484	No

Read Code	Read Code Type	Read Code Description	Map Type	Mapped ICD-10/OPCS-4 Code	Non-index event
Xafis	V3	Atrial fibrillation detected	ICD10	I489	No
79340	V3	Percutaneous transluminal ablation of atrioventricular node	OPCS4	K571	No
7A6A1	V3	Operation on the pulmonary venous system	OPCS4	K621	No
7L1H0	V3	Direct current cardioversion	OPCS4	X501	No
7L1H1	V3	External cardioversion NEC	OPCS4	X502	No
7L1H2	V3	Internal electrode cardioversion	OPCS4	K624	No
7L1H3	V3	Electrical sinus rhythm conversion	OPCS4	X501	No
7L1H4	V3	Electrical operative cardiac stimulation	OPCS4	X501	No
X011n	V3	Synchronised direct current defibrillation	OPCS4	X501	No
XE0Jj	V3	External cardioversion NEC	OPCS4	X502	No
XM1KN	V3	External electrode cardioversion	OPCS4	X501	No
Xa1nl	V3	Direct current cardiac shock	OPCS4	X501	No
Xa3ru	V3	Electrical cardioversion NOS	OPCS4	X501	No
XaBdb	V3	Direct current defibrillation	OPCS4	X501	No
XaLgF	V3	Percutaneous transluminal ablation of atrial wall	OPCS4	K575	No
XaLjD	V3	Operations on individual pulmonary veins	OPCS4	K621	No
XaLjI	V3	Other specified operations on individual pulmonary veins	OPCS4	K621	No
XaLjJ	V3	Operations on individual pulmonary veins NOS	OPCS4	K621	No
XaMmb	V3	Percutaneous transluminal internal cardioversion NEC	OPCS4	K624	No
XaMmc	V3	Percutaneous transluminal ablation of atrial wall for atrial flutter	OPCS4	K622	No
XaMmd	V3	Percutaneous transluminal ablation of pulmonary vein to left atrium conducting system	OPCS4	K621	No
XaMrA	V3	Percutaneous transluminal ablation of atrial wall NEC	OPCS4	K575	No
XaMrB	V3	Percutaneous transluminal ablation of conducting system of heart for atrial flutter NEC	OPCS4	K623	No
XaOfF	V3	Chemical cardioversion	OPCS4	X502	No
XaabV	V3	Percutaneous occlusion of left atrial appendage	OPCS4	K625	No
Xaasw	V3	Exclusion of left atrial appendage	OPCS4	K223	No
3272	V3	ECG: atrial fibrillation	KW	NA	No
3273	V3	ECG: atrial flutter	KW	NA	No
7936A	V3	Implantation of intravenous pacemaker for atrial fibrillation	KW	NA	No

Read Code	Read Code Type	Read Code Description	Map Type	Mapped ICD-10/OPCS-4 Code	Non-index event
XE0Wk	V3	(Atrial fibrillation) or (atrial flutter)	KW	NA	No
XaDv6	V3	H/O: atrial fibrillation	KW	NA	Yes
XaIIT	V3	Atrial fibrillation monitoring	KW	NA	Yes
XaLFh	V3	Exception reporting: atrial fibrillation quality indicators	KW	NA	No
XaLFi	V3	Excepted from atrial fibrillation quality indicators: Patient unsuitable	KW	NA	No
XaLFj	V3	Excepted from atrial fibrillation quality indicators: Informed dissent	KW	NA	No
XaLFz	V3	Atrial fibrillation resolved	KW	NA	Yes
XaMDF	V3	Atrial fibrillation monitoring administration	KW	NA	Yes
XaMDG	V3	Atrial fibrillation monitoring first letter	KW	NA	Yes
XaMDH	V3	Atrial fibrillation monitoring second letter	KW	NA	Yes
XaMDI	V3	Atrial fibrillation monitoring third letter	KW	NA	Yes
XaMDK	V3	Atrial fibrillation monitoring verbal invite	KW	NA	Yes
XaMFn	V3	Atrial fibrillation monitoring telephone invite	KW	NA	Yes
XaMGD	V3	Atrial fibrillation annual review	KW	NA	Yes
XaNRA	V3	History of atrial flutter	KW	NA	Yes
XaXrZ	V3	Referral to atrial fibrillation clinic	KW	NA	No
XaZdc	V3	Atrial fibrillation care pathway	KW	NA	No
XaaaD	V3	Provision of written information about atrial fibrillation	KW	NA	No

Mapped code refers to the ICD-10 or OPCS-4 code used to identify the relevant Read Code. Non-index events identify Read Codes which suggest a prior or historical diagnosis of AF. ICD-10 – international classification of disease 10 code, KW – keyword, NA – not applicable, OPCS-4 – OPCS classification of interventions and procedures version 4, V2 – Read Code version 2, V3 – Read Code version 3.

Supplementary Table 3. Country-specific record availability dates

	PC	HADP	Combined
England	31 May 2016	30 June 2020	31 May 2016
Scotland	31 March 2017	31 October 2016	31 October 2016
Wales	31 August 2017	29 February 2016	29 February 2016

Supplementary Table 4: Drug names, Read 2 codes, and BNF codes used to derive anticoagulation status in PC

Extraction term	Description	Drug Category
**BNF Codes**		
020801	Parental anticoagulants	-
020802	Oral anticoagulants	-
**Drug names**		
Acenocoumarol (Nicoumalone, Sinthrome)	-	VKA
Apixaban (Eliquis)	-	DOAC
Bemiparin (Zibor)	-	LMWH
Certoparin (Alphaparin)	-	LMHW
Dabigatran (Pradaxa)	-	DOAC
Dalteparin (Fragmin)	-	LMWH
Danaparoid (Orgaran)	-	Heparinoid
Desirudin (Revasc)	-	Heparinoid
Dicoumarol	-	VKA
Edoxaban (Lixiana)	-	DOAC
Enoxaparin (Clexane)	-	LMWH
Fondaparinux (Arixtra)	-	Heparinoid
Heparin (Calciparine, Monoparin, Multiparin, Minihep, Unihep, Uniparin)	-	Heparinoid
Lepirudin (Refludan)	-	Heparinoid
Phenindione (Dindevan)	-	VKA
Reviparin (Clivarine)	-	LMWH
Rivaroxaban (Xarelto)	-	DOAC
Tinzaparin (Innohelp, Logiparin)	-	LMWH
Warfarin (Marevan)	-	VKA
**Read 2 codes**		
bs...	Oral anticoagulants	-
br...	Parenteral anticoagulants	-
bs2.., bs23.	Acenocoumarol	VKA
br93., br94.	Alphaparin	LMWH
bs7.., bs72., bs74.	Apixaban	DOAC
brD5., brD6., brD7., brD9., brDZ.	Arixtra	Heparinoid
brE.., brE1., brE2., brE3., brE4., brE5.	Bemiparin	LMWH
br23., br24., br25., br2g.	Calciparine	Heparinoid
br9.., br91., br92.	Certoparin	LMWH
br63., br64., br68., br69., br6A., br6B., br6C., br6D.	Clexane	LMWH
brC1.	Clivarine	LMWH
bs4..,bs4x.,bs4y.,bs4z.	Dabigatran	DOAC
br2A.,br2C.,br2D.,br2E.,br2I.,br2l.,br2L.,br2m.,br2n.,br2o.,br2z.	Dalteparin	LMWH
br8.., br82.	Danaparoid	Heparinoid
brB.., brB1.	Desirudin	Heparinoid
bs5..	Dicoumarol	VKA
bs31.,bs32.,bs33.	Dindevan	VKA
bs8..,bs84.,bs85.,bs86.	Edoxaban	DOAC
bs71.,bs73.	Eliquis	DOAC
br6..,br61.,br62.,br65.,br66.,br67.,br6x.,br6y.,br6z.	Enoxaparin	LMWH
brD..,brD1.,brD2.,brD3.,brD4.,brD8.	Fondaparinux	Heparinoid
br1t.,br1u.,br2B.,br2F.,br2G.,br2h.,br2H.,br2i.,br2j.,br2J.,br2k.,br2K.,br2M.	Fragmin	LMWH

br1..,br11.,br12.,br13.,br14.,br15.,br16.,br17.,br18.,br19.,br1v.,br1w.,br2..,br21.,br22.,br2q.,br2r.,br2s.,br2t.,br2u.,br2v.,br2w.	Heparin	Heparinoid
br71.,br72.,br7A.,br7D.,br7H.,br7J.,br7K.,br7N.,br7O.,br7Q.,br7R.,br7T.,br7U.,br7V.	Innohep	LMWH
brA..,brA1.	Lepirudin	Heparinoid
bs81.,bs82.,bs83.	Lixiana	DOAC
br73.,br74.,br75.	Logiparin	LMWH
bs11.,bs12.,bs13.,bs1B.	Marevan	VKA
br26.,br27.,br28.,br29.,br2x.,br2y.	Minihep	Heparinoid
br1a.,br1A.,br1b.,br1c.,br1d.,br1q.,br1s.,br2a.	Monoparin	Heparinoid
br2e.	Monoparin-Ca	Heparinoid
br1e.,br1f.,br1g.	Multiparin	Heparinoid
bs24.	Nicoumalone	VKA
br81.	Orgaran	Heparinoid
bs3..,bs34.,bs35.,bs36.	Phenindione	VKA
bs41.,bs42.,bs43.	Pradaxa	DOAC
br1h., br1i., br1j.	Pump-Hep	Heparinoid
brA2.	Refludan	Heparinoid
brB2.	Revasc	Heparinoid
brC..,brCz.	Reviparin	LMWH
bs6..,bs6w.,bs6x.,bs6y.,bs6z.	Rivaroxaban	DOAC
bs22.,bs21.	Sinthrome	VKA
br7..,br76.,br77.,br78.,br79.,br7B.,br7C.,br7E.,br7F.,br7G.,br7L.,br7M.,br7P.,br7S.,br7W.,br7X.,br7Y.	Tinzaparin	LMWH
br1k.,br1l.,br1m.,br1n.	Unihep	Heparinoid
br2b.,br2d.,br2f.	Uniparin	Heparinoid
br2c.,br2p.	Uniparin-Ca	Heparinoid
bs14.,bs15.,bs16.,bs1..,bs17.,bs18.,bs19.,bs1A.,bs1C.	Warfarin	VKA
bs61.,bs62.,bs63.,bs64.	Xarelto	DOAC
brE6.,brE7.,brE8.,brE9.,brEA.	Zibor	LMWH

DOAC – direct oral anticoagulant, BNF – British National Formulary, LMWH – low molecular weight heparin, VKA – vitamin K antagonist. Generic drug names and brand equivalents (in brackets) were searched. Read2 codes are case specific.

Supplementary Table 5. Frequency of Read v2 and Read v3 codes used to identify 5,032 AF cases through PC records

Read code description	Frequency	%	Read v2 code	Read v3 code	
Atrial fibrillation	2904	57.7%	G5730	G5730	
Paroxysmal atrial fibrillation	953	18.9%	G5732	Xa2E8	
Atrial fibrillation and flutter	390	7.8%	G573.	G573.	
Atrial flutter	270	5.4%	G5731	G5731	
Atrial fibrillation monitoring	149	3.0%	662S.	XaIIT	*
Direct current cardioversion	58	1.2%	7L1H0	7L1H0	
H/O: atrial fibrillation	39	0.8%	14AN.	XaDv6	*
Atrial fibrillation resolved	31	0.6%	212R.	XaLFz	*
Referral to atrial fibrillation clinic	28	0.6%	8HTy.	XaXrZ	*
Atrial fibrillation annual review	26	0.5%	6A9..	XaMGD	
Percutaneous transluminal ablation of atrioventricular node	21	0.4%	79340	79340	
Atrial fibrillation monitoring administration	20	0.4%	9Os..	XaMDF	*
Paroxysmal atrial flutter	18	0.4%	G5736	XaaUH	
Rapid atrial fibrillation	18	0.4%		XaEga	
Persistent atrial fibrillation	17	0.3%	G5735	XaOfa	
Atrial fibrillation and flutter NOS	16	0.3%	G573z	G573z	
External cardioversion NEC	10	0.2%	7L1H1	7L1H1, XE0Jj	
Permanent atrial fibrillation	9	0.2%	G5734	XaOft	
Excepted from atrial fibrillation quality indicators: Patient unsuitable	7	0.1%	9hF0.	XaLFi	*
Percutaneous transluminal ablation of atrial wall for atrial flutter	7	0.1%	793M1	XaMmc	
Controlled atrial fibrillation	5	<0.1%		Xa7nI	
Electrical cardioversion NOS	5	<0.1%		Xa3ru	
History of atrial flutter	5	<0.1%	14AR.	XaNRA	*
Atrial fibrillation monitoring first letter	4	<0.1%	9Os0.	XaMDG	*
Lone atrial fibrillation	4	<0.1%		X202R	
Atrial fibrillation monitoring verbal invite	3	<0.1%	9Os3.	XaMDK	*
Percutaneous transluminal ablation of conducting system of heart for atrial flutter NEC	3	<0.1%	793M3	XaMrB	
Chemical cardioversion	2	<0.1%	7L1H8	XaOfF	
Percutaneous transluminal ablation of pulmonary vein to left atrium conducting system	2	<0.1%	793M0	XaMmd	
Atrial fibrillation care pathway	1	<0.1%	8CMW2	XaZdc	*
Atrial fibrillation monitoring telephone invite	1	<0.1%	9Os4.	XaMFn	*
Atrial fibrillation monitoring third letter	1	<0.1%	9Os2.	XaMDI	*
Direct current defibrillation	1	<0.1%		XaBdb	
Excepted from atrial fibrillation quality indicators: Informed dissent	1	<0.1%	9hF1.	XaLFj	*
Non-rheumatic atrial fibrillation	1	<0.1%	G5733	X202S	
Percutaneous transluminal ablation of atrial wall NEC	1	<0.1%	79348	XaMrA	

Percutaneous transluminal internal cardioversion NEC	1	<0.1%	793M2	XaMmb

* Read codes identified through key-word search.

Supplementary Table 6. Additional participant-level information

Characteristics	PC-only (n = 1,571)	PC + HADP (n = 3,461)	HADP-only (n = 2,104)
**Hospital admissions during follow-up**			
Total hospital admissions, median (IQR)	3.0 (1.0, 6.0)	7.0 (4.0, 11.0)	9.0 (5.0, 15.0)
Total hospital admissions with AF recorded, median (IQR)	0	2.0 (1.0, 3.0)	1.0 (1.0, 2.0)
Total admissions without AF recorded, median (IQR)	3.0 (1.0, 6.0)	4.0 (2.0, 8.0)	7.0 (3.0, 13.0)
**Hospital admissions subsequent to the first AF admission**			
Total subsequent hospital admissions, median (IQR)	0.00 (0.00, 1.00)	2.00 (0.00, 4.00)	1.00 (0.00, 4.00)
Total subsequent hospital admissions > 0, n (%)	524 (33.3%)	2,508 (72.5)	1,397 (66.4)
Total subsequent admissions with AF recorded, median (IQR)	0	1.00 (0.00, 2.00)	0.00 (0.00, 1.00)
Total subsequent admissions with AF recorded > 0, n (%)	0	2,126 (61.4)	688 (32.7)
**PC encounters during follow-up**			
Total PC encounters, median (IQR)	144.0 (97.0, 207.0)	185.0 (125.0, 261.0)	139.0 (46.8, 232.5)
Total PC encounters > 0, n (%)			
Total PC encounters with AF recorded, median (IQR)	0.0 (0.0, 1.0)	1.0 (0.0, 1.0)	0
Total PC encounters without AF recorded > 0, n (%)	1,571 (100.0)	3,461 (100.0)	2,093 (99.5)
**PC encounters subsequent to the first AF encounter**			
Total subsequent PC encounters, median (IQR)	33.0 (15.0, 62.0)	70.0 (36.0, 117.0)	19.0 (2.0, 55.0)
Total subsequent PC encounters > 0, n (%)	1,568 (99.8)	3,460 (100.0)	1,772 (84.2)
Total subsequent PC encounters with AF recorded, median (IQR)	0.0 (0.0, 1.0)	1.0 (0.0, 1.0)	0
Total subsequent PC encounters with AF recorded > 0, n (%)	729 (46.4)	2,060 (59.5)	0
**First hospital admission with AF recorded**			
Admission duration (days)	--	1.0 (0.0, 4.0)	4.0 (1.0, 10.0)
Diagnostic position of the first AF episode is primary*, n (%)		1,979 (57.2%)	391 (18.6%)
Ascertainment source of the first AF episode, n (%)			
ICD-10 only	--	3,011 (87.0%)	1,910 (90.8%)
OPCS-4 only	--	19 (0.5%)	101 (4.8%)

Both	--	431 (12.5%)	93 (4.4%)

* Episodes are defined as a period of continuous care from a single consultant, and admissions are (often) made up of multiple episodes. PC, primary; HADP, hospital admissions diagnoses and procedures, IQR, inter-quartile range (Q1, Q3).

Supplementary Table 7. Proportion of participants who were anticoagulated at 3,6,12 months and at any time after AF ascertainment

	Anticoagulated at:			
	**3 months**	**6 months**	**12 months**	**Any time during follow-up**
**Participants requiring anticoagulation**				
PC-only	253 / 572 (44)	283 / 571 (50)	310 / 567 (55)	349 / 574 (61)
PC + HADP	684 / 1,421 (48)	785 / 1,410 (56)	846 / 1,390 (61)	1,110 / 1,437 (77)
HADP-only	83 / 802 (10)	88 / 780 (11)	85 / 752 (11)	117 / 918 (13)
All	1,020 / 2,795 (36)	1,156 / 2,761 (42)	1,241 / 2,709 (46)	1,576 / 2,631 (60)

Numbers in cells are counts (%). Percentages reflect the proportion of individuals who were alive at the end of each window. Participants were deemed to require anticoagulation if their CHA2DS2-VA score was ≥2

Supplementary Table 8. Incidence of ischaemic stroke and death after AF ascertainment, by ascertainment group

Outcome	Group	Events	Person-years	Incidence rate (95% CI)		
**Sequential adjustment:**				**None**	**+ baseline risk factors & service use**	**+ Charlson index**
Ischaemic stroke	PC-only	7	4180	1.7 (0.8, 3.5)	1.5 (0.7, 3.2)	1.6 (0.7, 3.3)
	PC + HADP	61	9748	6.3 (4.9, 8.0)	5.6 (4.2, 7.4)	5.8 (4.4, 7.7)
	HADP-only	38	5193	7.3 (5.3, 10.0)	6.5 (4.6, 9.1)	6.3 (4.5, 9.0)
Cardiovascular death	PC-only	9	4196	2.1 (1.1, 4.1)	1.9 (1.0, 3.7)	1.9 (1.0, 3.7)
	PC + HADP	75	9882	7.6 (6.0, 9.5)	5.6 (4.3, 7.3)	6.1 (4.7, 7.9)
	HADP-only	98	5269	18.4 (15.1, 22.4)	12.5 (9.8, 16.0)	11.3 (8.7, 14.5)
Non-cardiovascular death	PC-only	26	4196	6.2 (4.2, 9.1)	5.2 (3.5, 7.8)	5.3 (3.5, 7.8)
	PC + HADP	155	9882	15.7 (13.4, 18.3)	13.0 (10.9, 15.5)	13.4 (11.3, 16.0)
	HADP-only	321	5269	60.9 (54.6, 67.9)	48.3 (42.3, 55.1)	31.8 (27.2, 37.2)
All-cause death	PC-only	39	4196	9.3 (6.8, 12.7)	8.6 (6.3, 11.9)	8.9 (6.5, 12.2)
	PC + HADP	231	9882	23.4 (20.5, 26.6)	20.2 (17.5, 23.2)	21.6 (18.8, 24.8)
	HADP-only	429	5269	81.2 (73.8, 89.2)	64.7 (57.8, 72.4)	47.9 (42.1, 54.4)

Based on poisson models with time of risk used as an offset. Time at risk begins at AF ascertainment (second AF ascertainment for PC+HADP individuals). Baseline risk factors include age, sex, race (white vs non-white), Townsend Deprivation Index, body mass index, current smoker, current drinker, hypertension, type 2 diabetes, heart failure, myocardial infarction. Service use includes the total number of hospital admissions and primary care encounters during follow-up. The Charlson index includes myocardial infarction, congestive heart failure, peripheral vascular disease, cerebrovascular disease, dementia, chronic pulmonary disease, connective tissue disease, peptic ulcer disease, mild liver disease, moderate or severe liver disease, diabetes without complications, diabetes with end organ damage, hemiplegia or paraplegia, renal disease, malignancies excluding neoplasm of skin, metastatic solid tumour, and AIDS/HIV. Models with ischaemic stroke as the outcome excluded heart failure as there was no prevalent heart failure in those with post-AF ischaemic stroke.

Supplementary Figure 1. Flowchart illustrating the process of mapping the HADP-based AF phenotype to primary care data

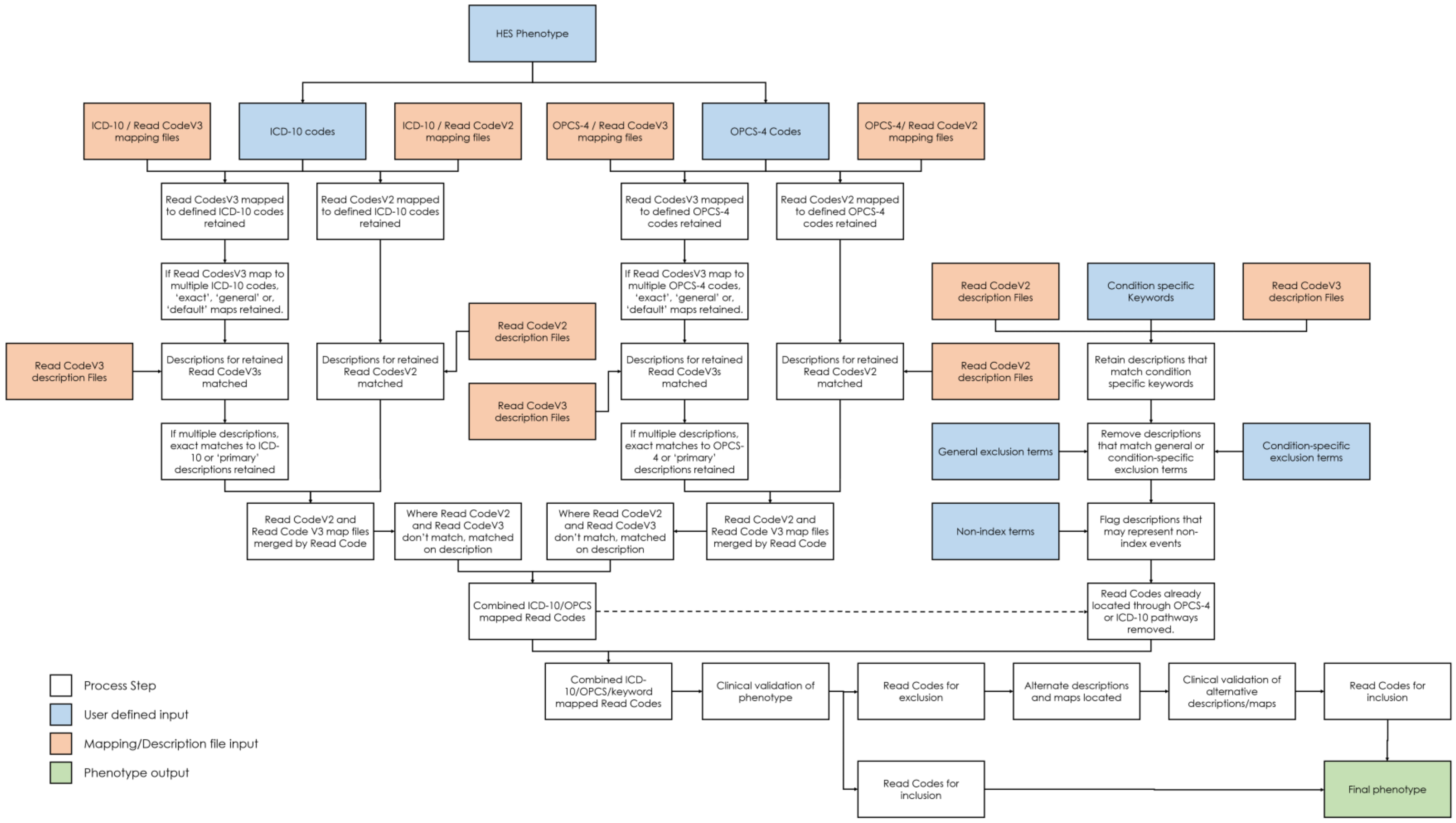

Supplementary Figure 2. Flowchart of included participants

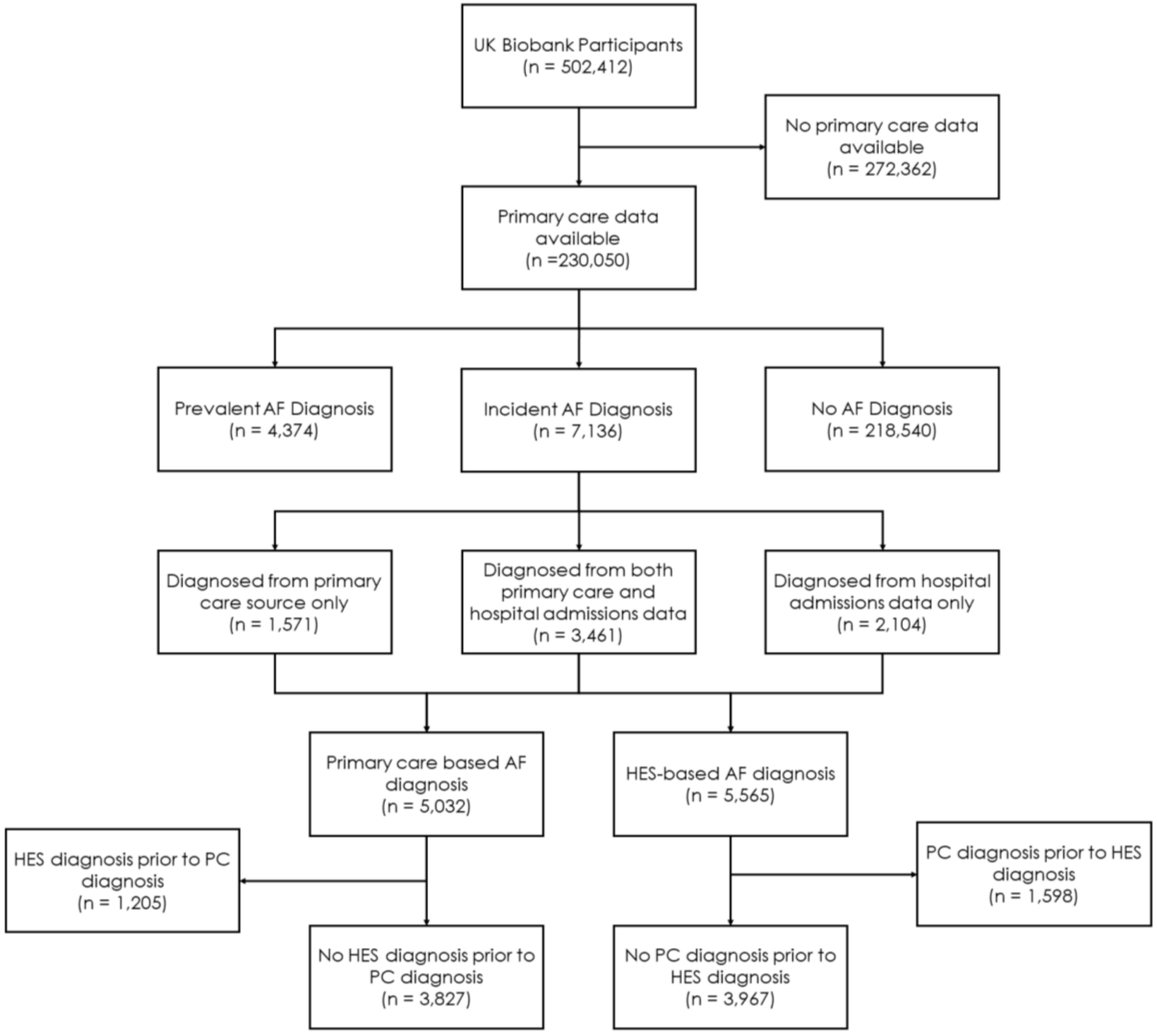

Supplementary Figure 3. Source of prevalent AF ascertainment

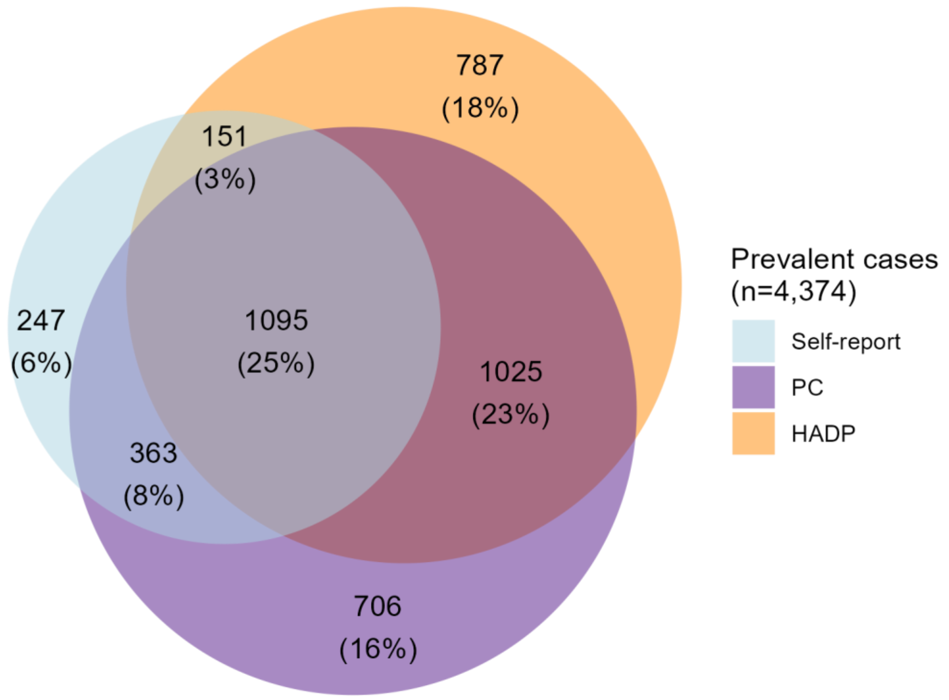

Percentages are percentage of total AF cases. HADP, hospital admissions diagnoses and procedures

Supplementary Figure 4. Time from PC to HADP AF ascertainment, by sex and age

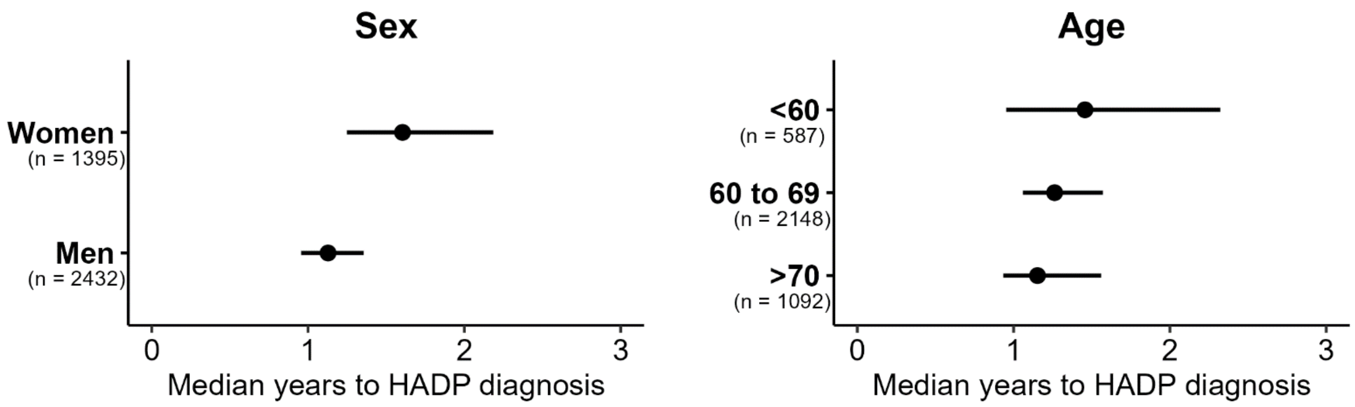

Median time to subsequent HADP ascertainment for cases identified through primary care, by age and sex. Median values were estimated using Kaplan Meier curves, with 95% confidence intervals estimated using the Brookmeyer-Crowley method.

Supplementary Figure 5: Time from PC to HADP AF ascertainment, by diagnostic code position (AF primary or non-primary)

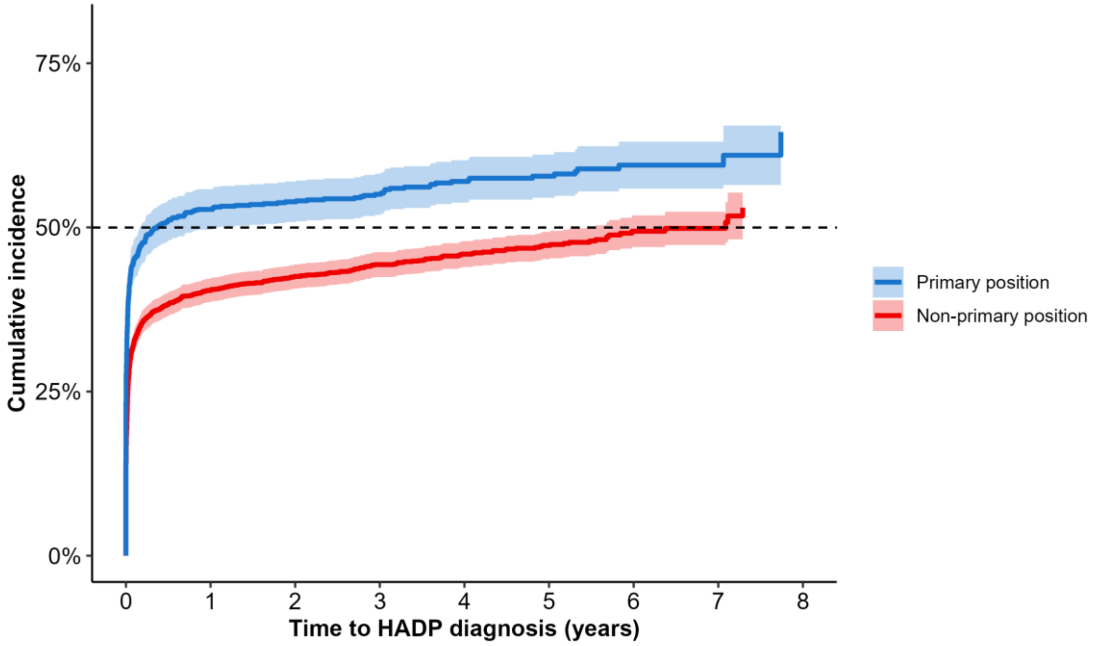

Supplementary Figure 6: Anticoagulation rate at 90 days by year and ascertainment group

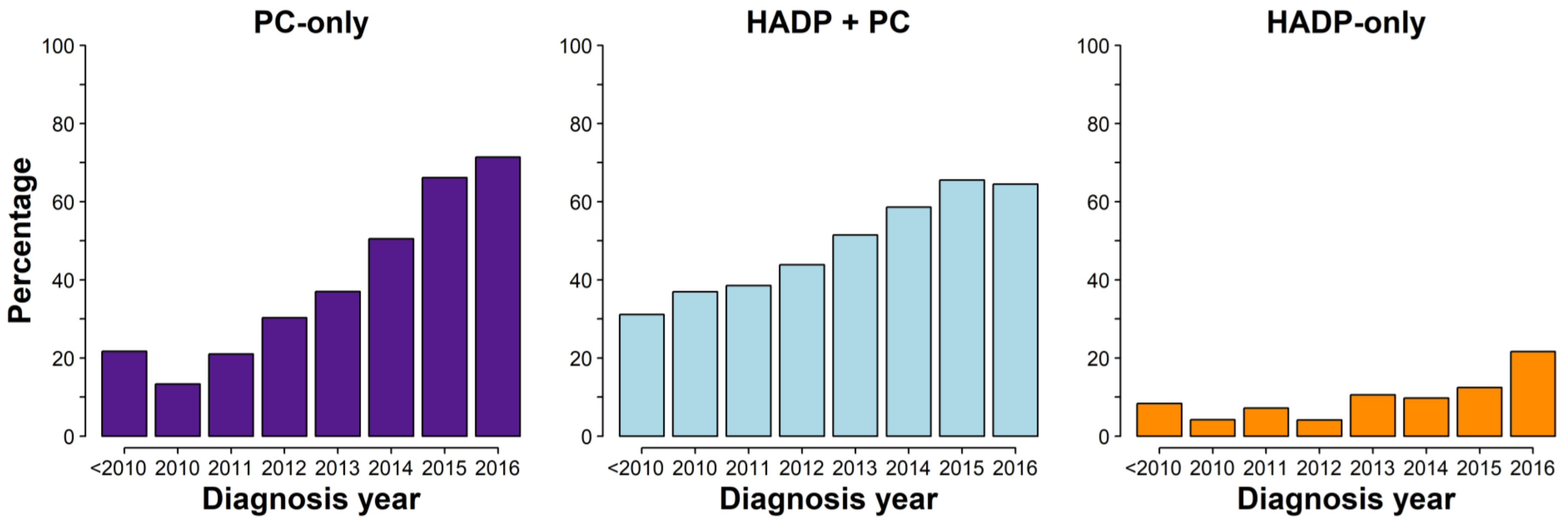

Bar plot demonstrating the proportion of participants who are anticoagulated within 90 days of record. Limited to participants with a baseline CHA2DS2-VA score ≥2 where oral anticoagulation would be recommended. Participants divided by year and AF ascertainment group.

Supplementary Figure 7: Anticoagulant drug class initiated by year and AF ascertainment group

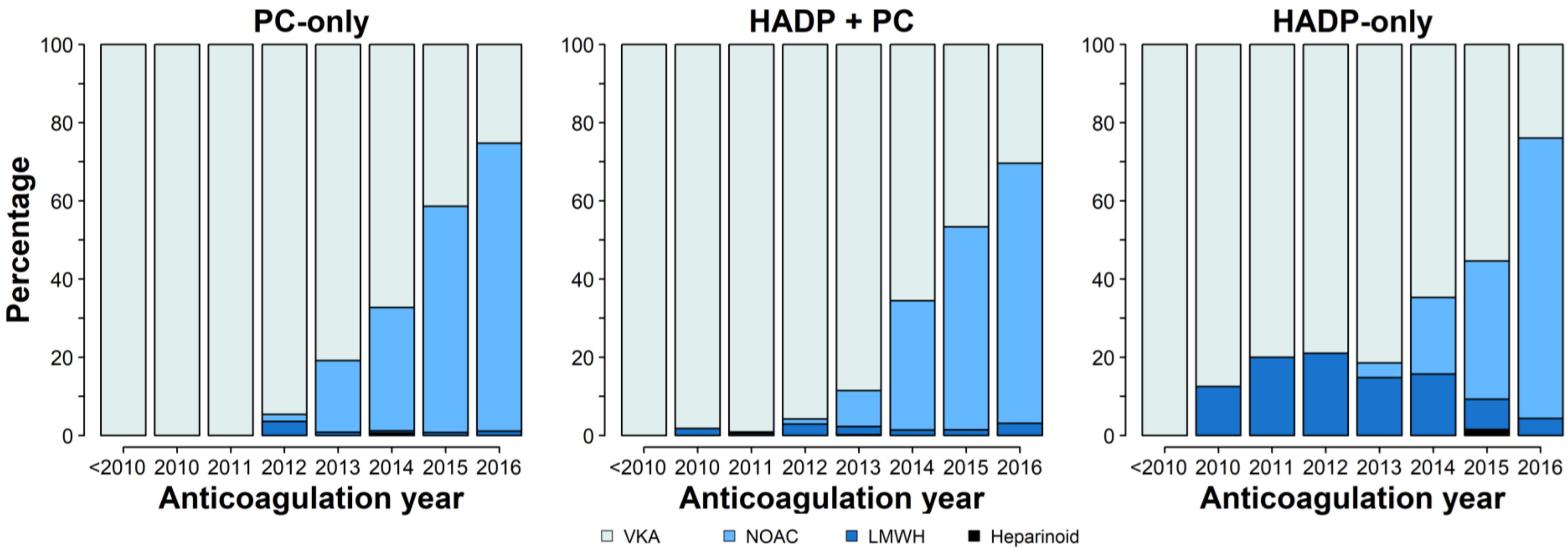

Bar plot demonstrating the proportion of each anticoagulant drug class first initiated in participants with an AF record by year of record and ascertainment group. The year of anticoagulation is defined as the first time in which a participant has both a prescription for anticoagulation and a record of AF. LMWH – low molecular weight heparins, NOAC – non-VKA oral anticoagulants, VKA – vitamin K antagonists.

Supplementary Figure 8: Time to anticoagulation grouped by participant sex and stratified by AF ascertainment group

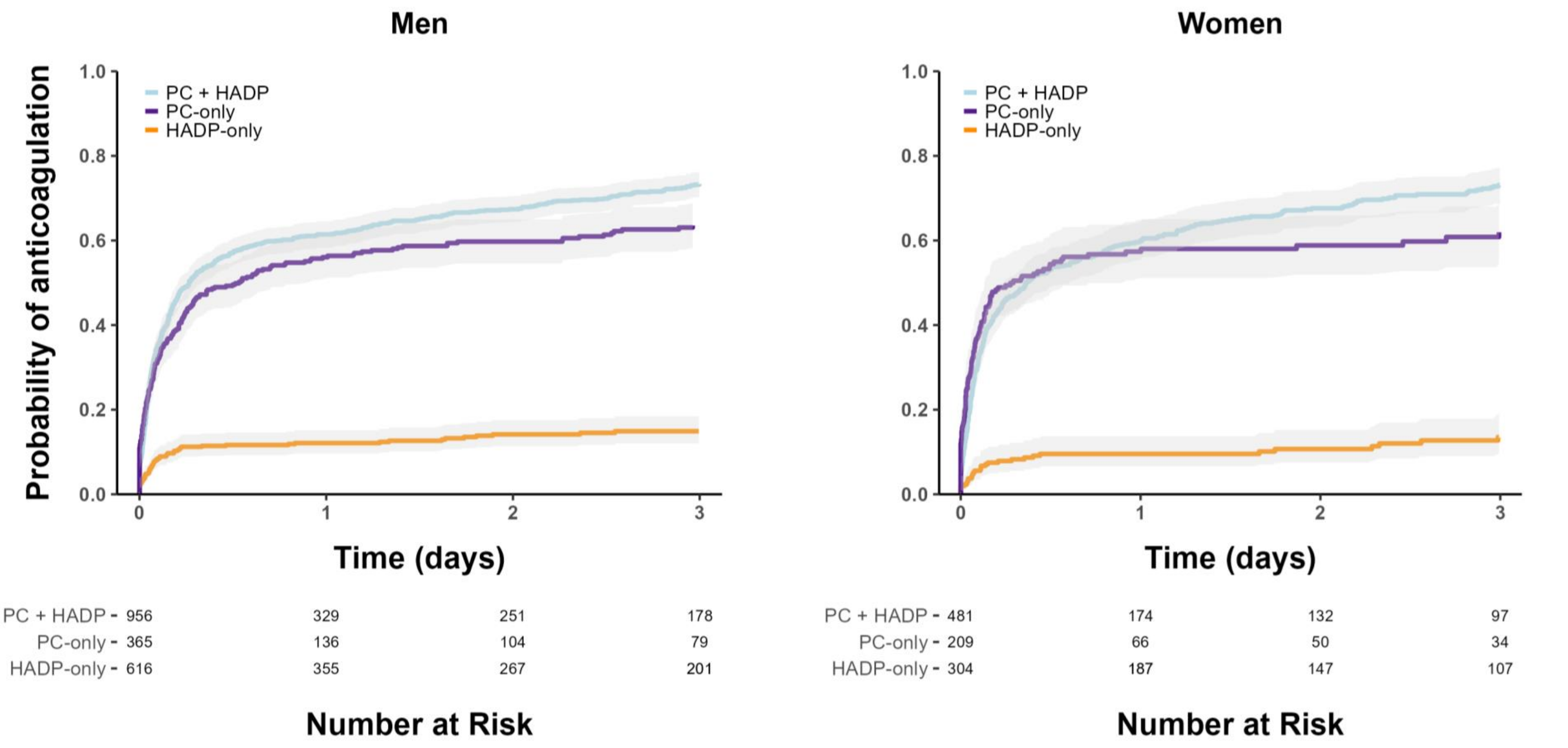

Cumulative incidence curves showing years to first oral anticoagulation prescription after AF ascertainment split by participant source, stratified by ascertainment group. Limited to participants with a baseline CHA2DS2-VA score ≥2 where oral anticoagulation would be recommended.

Supplementary Figure 9: Time to anticoagulation grouped by date of AF ascertainment and stratified by ascertainment group

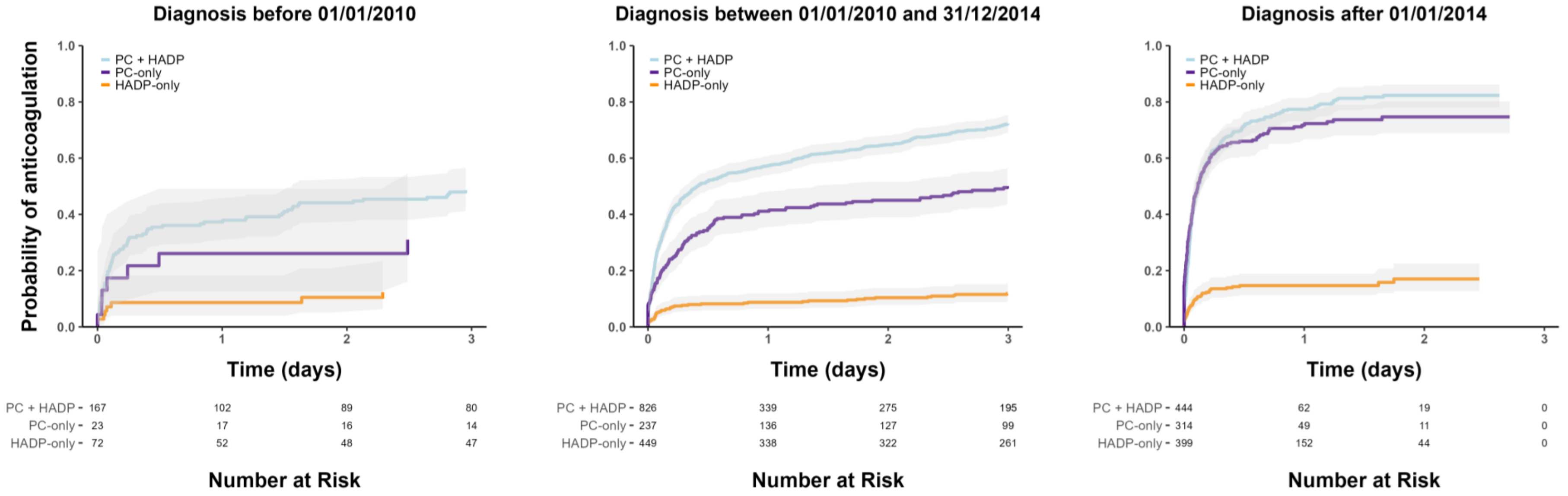

Cumulative incidence curves showing years to first oral anticoagulation prescription after AF ascertainment split by date of AF record, stratified by ascertainment group. Limited to participants with a baseline CHA2DS2-VA score ≥2 where oral anticoagulation would be recommended.

Supplementary Figure 10: Time to anticoagulation for individuals with a Charlson comorbidity index of 0 at AF ascertainment, stratified by ascertainment group

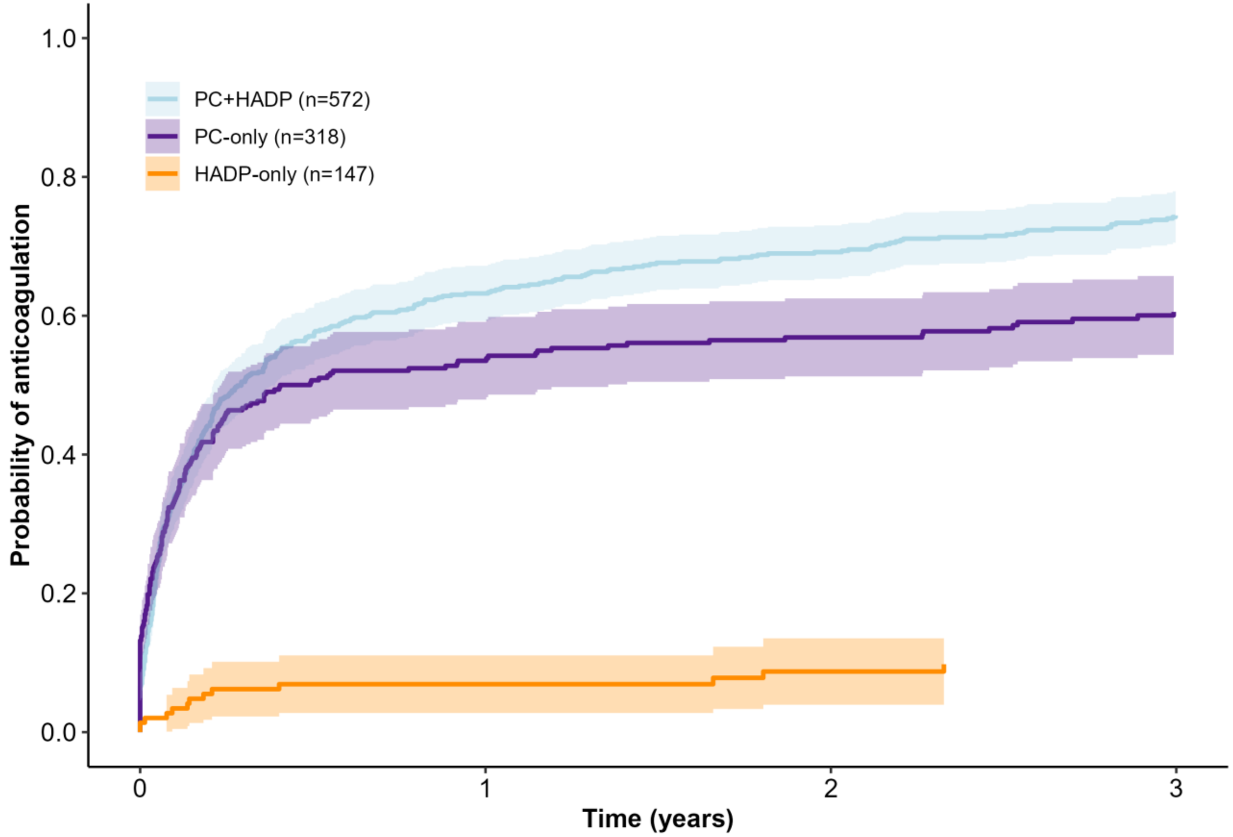

Cumulative incidence curves showing years to first oral anticoagulation prescription after AF ascertainment for individual with a Charlson comorbidity score of zero at AF ascertainment, stratified by ascertainment group. Limited to participants with a baseline CHA2DS2-VA score ≥2.

.

Supplementary Figure 11: Time to anticoagulation for individuals with AF recorded as the primary cause of admission in the hospital record, stratified by ascertainment group

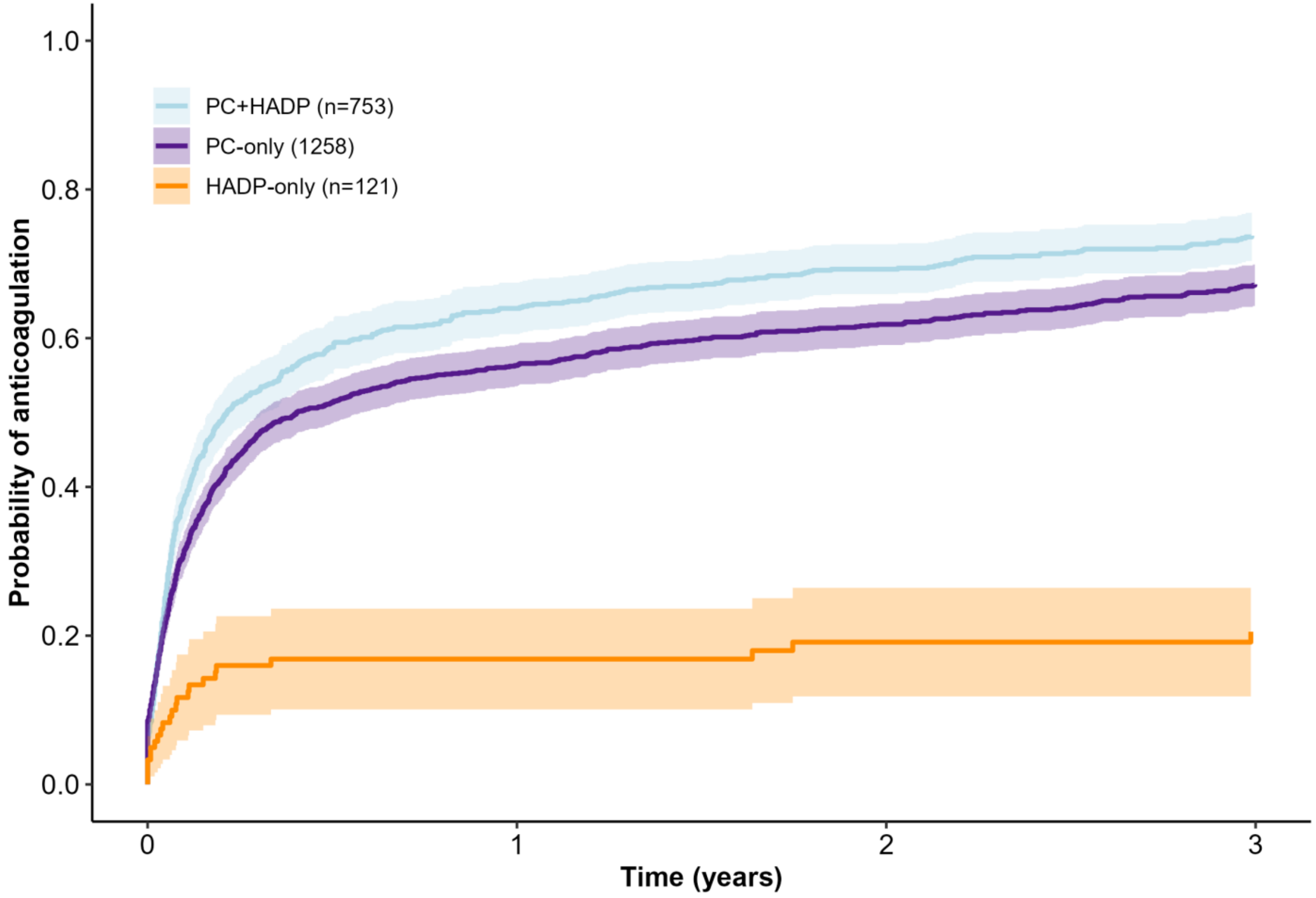

Cumulative incidence curves showing years to first oral anticoagulation prescription after AF ascertainment, where only AF recorded in the primary diagnostic position in the hospital record (i.e. the primary cause of hospital admission) was used to define HADP-based AF cases.
